# Qualitative and quantitative dermatoglyphics of chronic kidney disease of unknown origin (CKDu) in Sri Lanka

**DOI:** 10.1186/s40101-019-0207-0

**Published:** 2020-01-17

**Authors:** Buddhika Thilanga Bandara Wijerathne, Robert John Meier, Sujatha Senadeera Salgado, Suneth Buddhika Agampodi

**Affiliations:** 1 Department of Community Medicine, Faculty of Medicine and Allied Sciences, Rajarata University of Sri Lanka, Saliyapura, 50008 Sri Lanka; 2 Department of Anthropology, Indiana University, Bloomington, IN USA; 3 Department of Anatomy, Faculty of Medicine, University of Kelaniya, Ragama, Sri Lanka

**Keywords:** Chronic kidney disease, Dermatoglyphics, Fluctuating asymmetry, Prenatal stress, Sri Lanka

## Abstract

**Background:**

Dermatoglyphics has been used widely in fields of medicine as a non-invasive diagnostic tool and an early assessment of risk for certain medical conditions. It reflects disturbances in fetal development during early prenatal weeks 14–22 when fingerprints develop. Dermatoglyphic asymmetry has been used to measure developmental instability during a specific period of human fetal development. Thus, the present study was planned to investigate whether digital and palmar dermatoglyphics of chronic kidney disease of unknown origin (CKDu) patients in Sri Lanka are different from healthy people.

**Methods:**

A case control study was carried out among CKDu patients (90 males, 90 females) from a CKDu endemic area and gender-matched two control groups; one group from a CKDu endemic region (90 males, 90 females) and another group from a CKDu non-endemic region (90 males, 90 females). Dermatoglyphics were obtained using photographic methods. Both qualitative and quantitative dermatoglyphic variables were defined and analyzed according to standard criteria. Both directional (DA) and fluctuating asymmetry (FA) were assessed.

**Results:**

Several qualitative dermatoglyphic variables had significant association with CKDu. The triradii a^1^ variable was less evident in palms of CKDu cases in both genders when compared to both control groups. The FA of pattern discordance (right vs left hands) between CKDu cases and control group were significant in several digits. The FA of the ridge count was found significant in several digits, and also significant for A-B ridge count and total ridge count.

**Conclusion:**

Based on these results, it is proposed that the mechanisms responsible for the development of CKDu might be associated with those responsible for FA observed in CKDu patients. Accordingly, a diagnostic tool based on FA could be developed for predicting risk prior to the development of CKDu.

## Background

Chronic kidney disease (CKD) is a global public health problem and progressively more common in developed as well as developing nations [1]. According to a Global Burden of Disease study in 2016, the age-standardized death rate for CKD was 18.2 per 100,000 people and disability-adjusted life-years (DALYs) was 473.9 [2]. The leading underlying risk factors for CKD are diabetes and hypertension [2]. However, a devastating form of CKD has been observed recently in several regions including Sri Lanka, Central America, India, and Egypt which was not attributed to conventional risk factors [3]. It has been referred to as CKD of unknown etiology (CKDu), and recent evidence suggested causative factors could be of environmental and/or occupational origin [4]. Due to the epidemiological pattern and histopathological similarities of the disease, a new entity called chronic interstitial nephropathy of agricultural communities (CINAC) was proposed to identify this condition [4, 5]. Despite extensive investigations, its exact etiology is still a mystery.

Dermatoglyphics is the study of epidermal ridge patterns (fingerprints) on the skin of the fingers, palms, toes, and soles that commence during embryological development between the sixth and seventh week of intrauterine life, and are fully formed by the 21st week [6]. It has been used widely in fields of anthropology, genetics, and medicine and as a valuable non-invasive diagnostic tool and early assessment of risk for certain medical conditions [7]. The relationship between different dermatoglyphic traits and various medical diseases have been widely evaluated, and the main hypothesis for support of this association is “if growth of the limbs is disturbed in very early fetal life, changes in the epidermal ridge configurations are likely” [8–10]. It should be added, however, that both environmental and genetic factors do influence the development of dermatoglyphics [6, 10–13]. Wilms’ tumor (WT) is the most common childhood renal tumor, and Curró et al. [14] study showed a significantly lower incidence of radial loops and whorls in WT patients. Gutjahr et al [15], showed a lower occurrence of digital arch patterns in affected cases and a slightly higher frequency of whorls in WT patients. A recent systematic review revealed an association of both qualitative and quantitative dermatoglyphic traits with several kidney diseases [7]. However, it was concluded that methodological issues may limit any interpretation of significant findings [7].

Symmetry is universal in the living world, and organisms display bilateral symmetries at every turn [16]. The extent to which the average individual departs from perfect symmetry is fluctuating asymmetry (FA) [17]. More specifically, FA has been defined as random differences between the right (R) and left (L) sides of a morphological trait [18]. Values of the right minus their corresponding values on the left are in fairly normal distribution with a mean of zero or close to zero, and any variance in the distribution of R-L difference is a measure of FA [16–19]. For distinction, directional asymmetry (DA) shows a significant departure from zero in the mean of R-L differences [16, 17]. Furthermore, anti-symmetry has a zero mean (or close to it) although the distribution around the mean is either platykurtic or bimodal. In essence, FA is then a population level measure of developmental instability, robustness, and developmental noise that is influenced by genetic factors along with their interaction with environmental stressors [17, 20–22]. Based upon these considerations, dermatoglyphic asymmetry can be used to measure developmental instability during a specific period of human fetal development [17]. Fluctuating asymmetry has been seen in dermatoglyphics as primarily concerned with the impact of environmental stress or noise that disrupts usual genetic expression [20, 22, 23].

We hypothesized that, if CKDu is partly due to a prenatal environmental exposure among genetically predisposed individuals, it might be associated with epidermal ridge formation and possibly lead to dermatoglyphic asymmetry. Thus, the present study was planned to investigate whether digital and palmar dermatoglyphics of CKDu patients in Sri Lanka are different from healthy people.

## Methods

### Study design

A case control study was carried out among CKDu patients from a CKDu endemic area and gender-matched two control groups; one group was selected from CKDu endemic region and another group from CKDu non-endemic region in Sri Lanka during 2014 to 2016. Ethical clearance was obtained from the Ethics Review Committee of Faculty of Medicine and Allied Sciences, Rajarata University of Sri Lanka (ERC/2013/31). Informed written consent to participate was obtained from all participants.

### Study settings

#### Cases

**Fig. 1:**
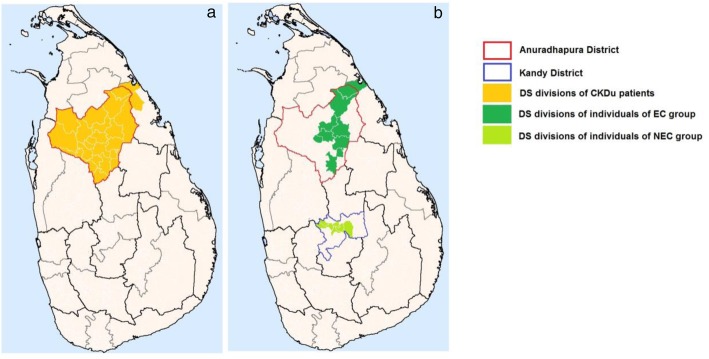
Geographical locations of cases (**a**) and controls (**b**)

The highest number of CKDu cases are reported from the Anuradhapura district in North Central Province (NCP) of Sri Lanka and considered as endemic for CKDu [24]. CKDu patients for the current study were selected from two CKDu clinics; kidney research and treatment unit of teaching hospital Anuradhapura and base hospital Padaviya (Fig. 1a). These patients were from all divisional secretariat (DS) divisions of the Anuradhapura districts, and a few patients were from the Adjacent Sri pura DS division in the eastern province.

#### Control groups

Community-based control groups were selected from both CKDu endemic (Anuradhapura District) and non-endemic (Kandy District) regions (Fig. 1b). Endemic controls (EC) were recruited from 9 DS divisions from Anuradhapura district while non-endemic controls (NEC) were recruited from 7 DS divisions in Kandy districts.

### Participant selection

All participants in cases and controls lived in their specified region for more than 10 years and belonged to the Sinhalese ethnic group for two generations.

#### Case definition

Cases included all CKDu patients that were diagnosed and followed up in two selected renal clinics. A medical doctor obtained the history and reviewed all medical records of CKDu patients and included only those patients who fulfilled the following criteria [24].

- No past history of glomerulonephritis, pyelonephritis, renal calculi
- Not on treatment for diabetes or (normal glycosylated hemoglobin (HbA1c); < 6.5%, normal fasting blood sugar)
- If on treatment for hypertension, blood pressure below < 140/90 mmHg; if not on treatment for hypertension, blood pressure below < 160/100 mmHg.

We also excluded patients with a past history of snake bites when envenomation required hospital admission for Russell’s viper and cobra bites.

#### Control groups

A medical doctor obtained a detailed history and examination, and participants were excluded if they were found to have a past history of:

- Kidney diseases (glomerular, tubular interstitial and cystic)
- Hypertension or on treatment for hypertension
- Diabetes or on treatment for diabetes
- History of urinary infection or infectious disease affecting kidney (leptospirosis or schistosomiasis)
- Prolong use of analgesics
- Snake bite with envenomation, required hospital admission for Russell’s viper and cobra bites

If participants were found to a have systolic blood pressure > 120 mmHg or diastolic blood pressure > 90 mmHg, they were reexamined after a 30-min rest, if they still had high BP they were not included.

In addition, the following laboratory investigations were also carried out for control selection.

- Estimated glomerular filtration rate (eGFR) based on CKD-EPI equation (> 60 mL/min/1.73m^2^) (If participant with eGFR between 60 and 90, urine albumin to creatinine ratio (ACR) and UFR values were checked for any abnormality and excluded if values were positive)
- ACR (< 30 mg/g)
- Normal UFR (without red cell cast)
- HbA1C < 6.5%

The palms and fingers of both hands of CKDu patients and healthy controls were examined during initial screening to exclude any participant with a medical condition such as psoriasis or significant scaring that altered the epidermal ridge pattern.

### Sample size calculation

The sample size was calculated by Kelsey formula for case control studies in OpenEpi software version 3.1 (OpenEpi, Atlanta, GA, USA) with *α*  =  0.05, power  =  80, ratio of cases to controls  =  1.0, hypothetical proportion of exposure among controls  =  50, and an odds ratio (OR) of 2 as a minimum difference between groups to be detected. This yielded a minimum sample size of 138 cases and 138 controls.

### Obtaining fingerprints

Participants were asked to wash their hands with soap and water thoroughly to remove dirt and dry them before obtaining fingerprints. If hands were moist, each finger was wiped with rubbing alcohol and then allowed to dry to avoid light reflection of the recorded image.

First, all fingers were examined for level 1 details (patterns) by principle investigator. We used a newly developed photographic method of fingerprint recording to avoid participant discomfort and to prevent a low quality of recording associated with the conventional ink-based method. We used a Canon EOS 60D camera with a Canon EF-S 18-135 mm f/3.5–5.6 IS STM lens. Images of digit D2 (index finger) to D5 (little finger) were initially obtained while placing the hand on a stage at a right angle. Then, both palms were photographed while resting them on a stage right angles to camera. Finally, thumbs were photographed at a right angle while keeping them in an upright position. If there were extra-limital patterns, each finger or palm was photographed from their sides. All images for each participant were checked on the camera screen to avoid any recording of out of focus (blurred) images, and the procedure was repeated if necessary.

**Fig. 2:**
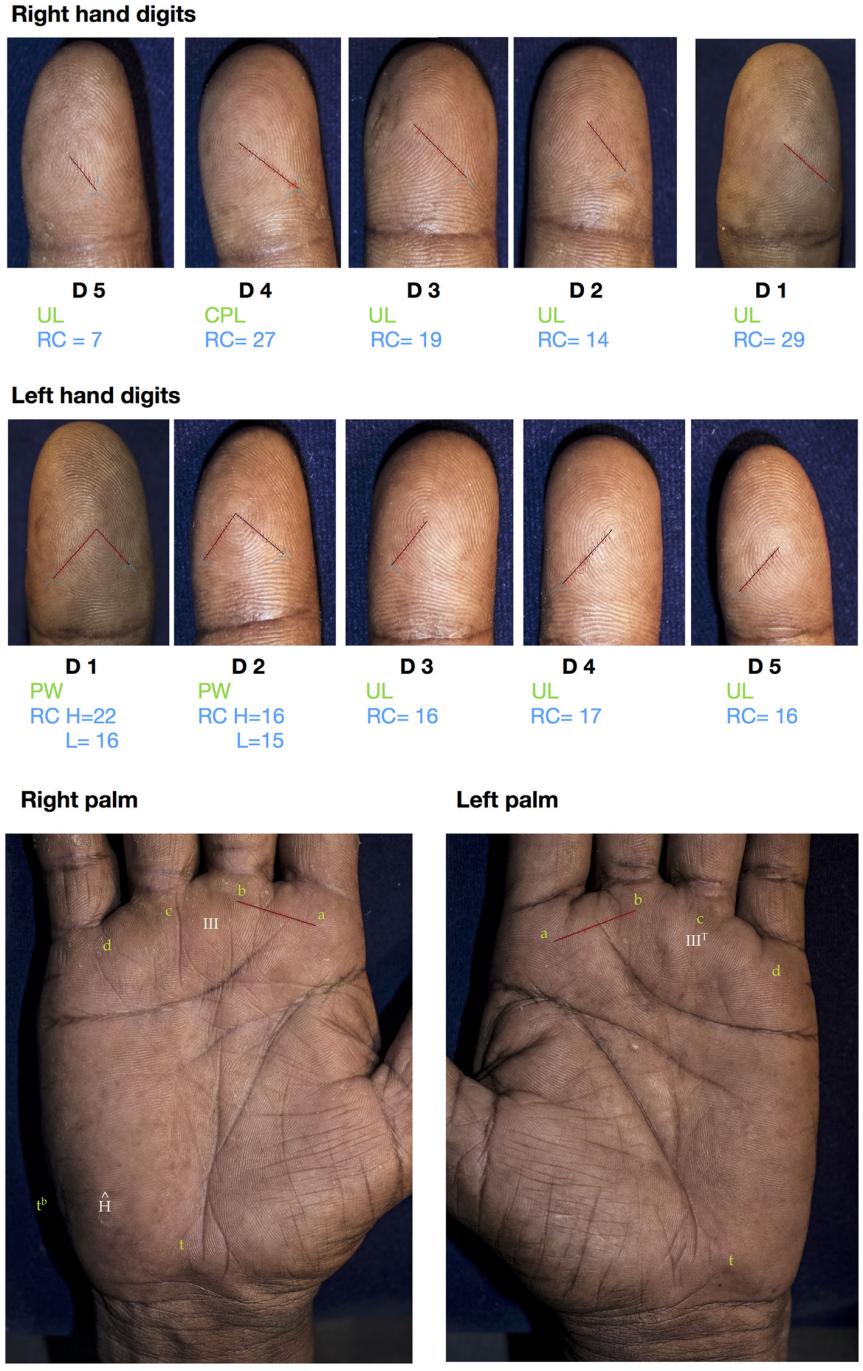
Plates with all digits and both palms of a person who has undergone analysis: **D** Digit. **UL** Ulnar loop. **CPL** Central pocket loop. **PW** plain whorl. **RC** ridge count. **H** higher count. **L** lower count.

All images were stored securely in two separate hard drives. Firstly, all ten fingers of the images were screened for clarity. Then, the RAW images were opened within Canon’s Digital Photo Professional software and adjustments were made on brightness, white balance, contrast, gamma correction, and sharpness. These adjustments allowed clear visibility of ridges of the recorded prints. Then images were opened through Microsoft Paint software, and each finger pattern area was cropped. These were arranged in a single file and saved as TIFF (Tagged Image File Format) (> 300 DPI) (Fig. 2) to preserve the high-quality data.

### Dermatoglyphics classification

**Fig. 3:**
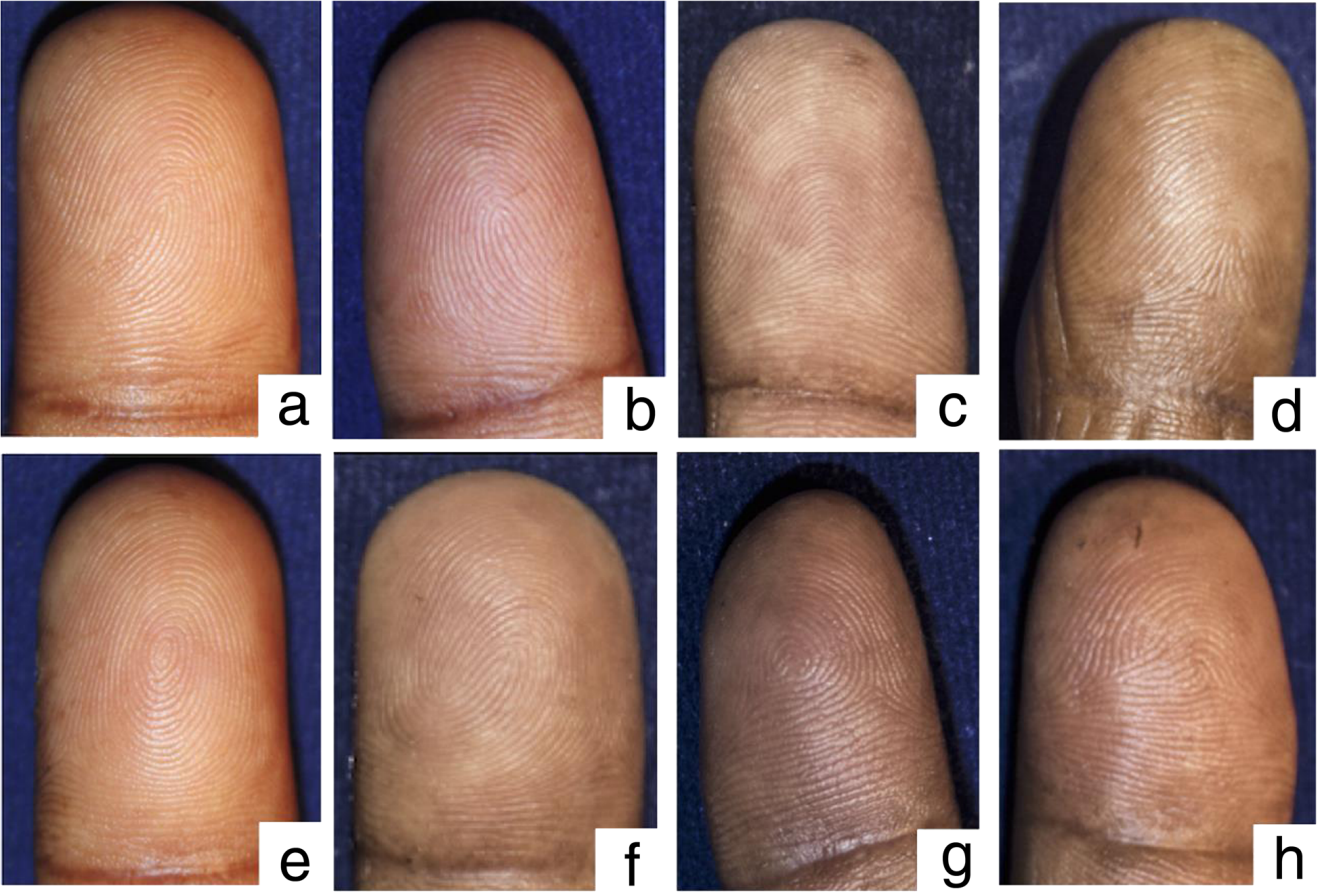
The digital fingerprint patterns: **a** Ulnar loop. **b**: Radial loop. **c** Plain arch. **d** Tented arch. **e** Plain whorl. **f** Double loop. **g** Central pocket loop. **h** Accidental whorl

The digital fingerprint patterns were classified into eight types: ulnar loop (UL), radial loop (RL), plain arch (PA), tented arch (TA), plain whorl (PW), double loop (DL), central pocket loop (CPL), and accidental whorl (A), according to the pattern classification described by the Federal Bureau of Investigation, USA (Fig. 3) [25]. Any pattern that could not be classified was marked as undefined. Patterns that were in severely scared areas where details could not be observed or where amputation had occurred were marked as missing.

**Fig. 4:**
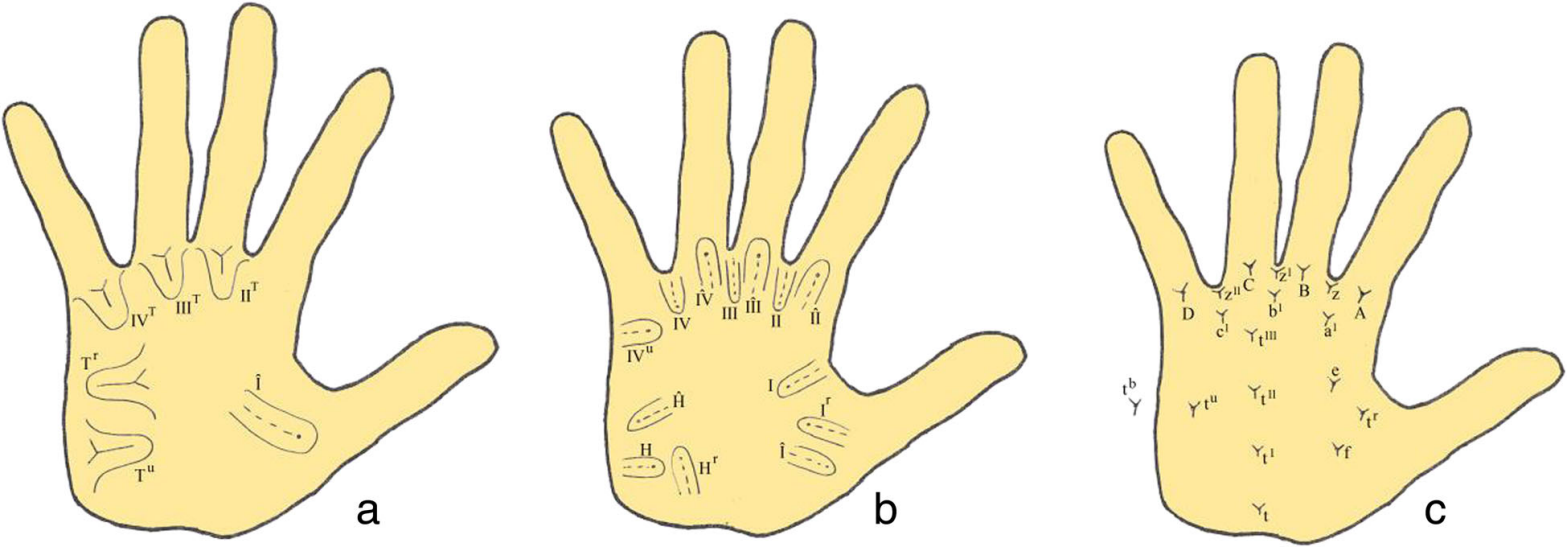
Palmar dermatoglyphics variables: **a**, **b** Loop patterns. **c** Triradii

Palmar dermatoglyphics variables were classified according to the Penrose Topological classification (Fig. 4) [11, 26]. Loops are specified according to the configuration area in which it occurs along with the direction of its core [26]. The configuration areas roughly correspond to fetal mounds [26]. Only true patterns, loops, and triradii are included in the description [11, 26]. Loops are designated by roman numerals according to the configuration area in which they were located and to the main direction of their cores, either distal (peripheral) or proximal (central) [11, 26]. Triradii were termed according to letters of the English alphabet [11, 26].

### Ridge counting and pattern intensity

**Fig. 5:**
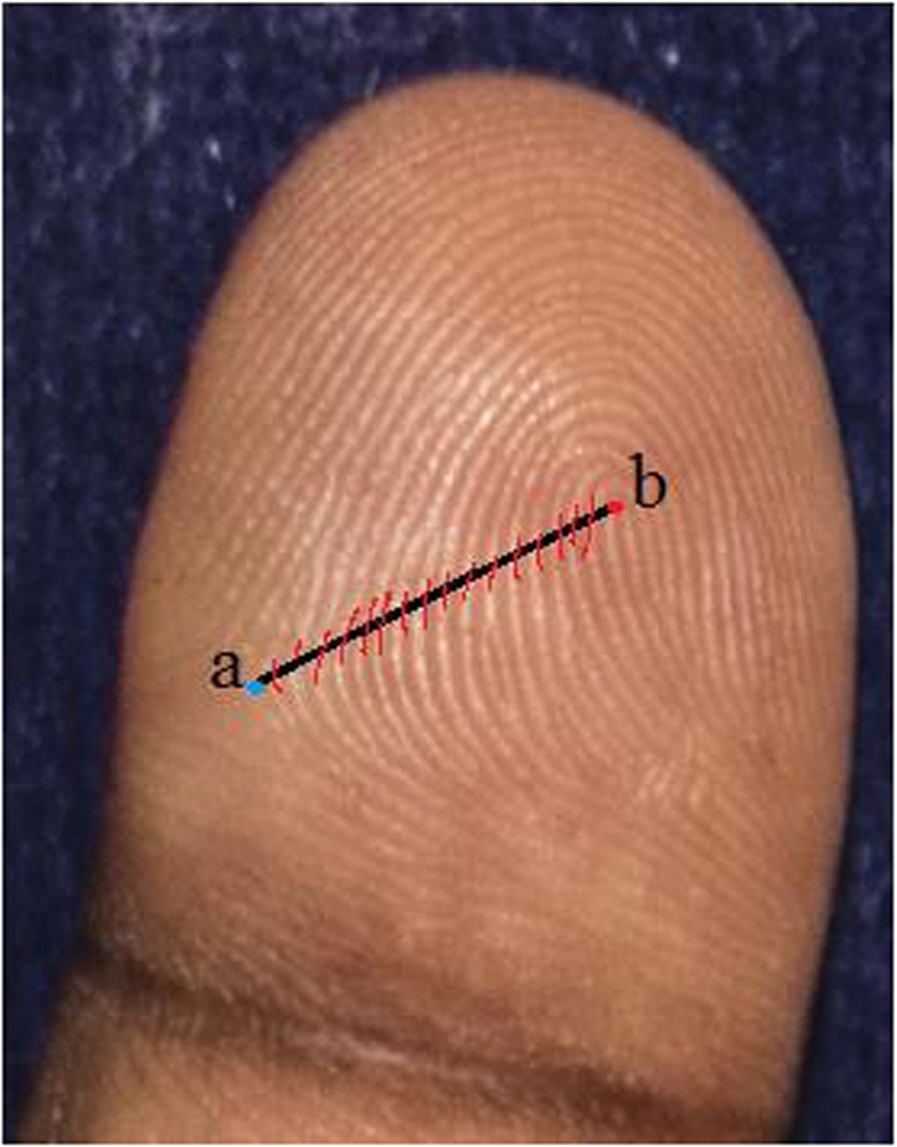
Finger ridge counting: **a** Triradius. **b** Core

Finger ridge count was defined as the number of ridges which intersect or touch a straight line drawn from the central point of a triradius to the center or core of the adjacent pattern [11]. Two ridges that result from a bifurcation of a single epidermal ridge and both cross the straight line are counted (Fig. 5). Any ridges that were close to the straight line without touching it were excluded [11]. Loop patterns have one ridge count while whorls usually have two ridge counts. Arches and other similar configurations that are not true patterns have a zero ridge count. When there was a missing finger on one hand, the ridge count on the corresponding finger of the other hand was inserted based on the considerable symmetry for this trait (this was done for three fingers in cases, two fingers in EC, one in NEC) [11].The sum of the largest ridge count on all ten fingers was defined as total ridge count (TRC) [11]. On the palm, the number of ridges that crossed a straight line connecting triradii “A” and “B” was defined as the A-B ridge count (A-B RC) [11].The pattern intensity index (PII) was calculated using the formula: PII = {(2 × % whorl + % loop) ÷ 10} [27]. PII essentially refers to the complexity of finger patterns in a specified population [6].

### Statistical analysis

The main demographic and clinical data were summarized using descriptive statistics.

The measure of central tendency used was the mean, and the measure of spread used was standard deviation (SD). Digital patterns were presented separately for each finger. The frequencies of the patterns were compared between the two groups using the chi-square test or the Fisher exact probability test. A *P* value of < 0.05 was considered statistically significant. All data were analyzed using GraphPad Prism 7.04 and SPSS® Version 20.

#### Directional asymmetry (DA) of Dermatoglyphics

The main aim of assessing DA is to identify whether one side was significantly larger than the other on average. We used the factorial ANOVA test to find out if significant differences existed between mean R and mean L of each homologous finger in a sample of individuals relative to the between-sides variation after accounting for measurement error. It also tested the significance of no DA and overall trait size variation among individuals. Antisymmetry artificially inflates the values of all FA indices. If a trait exhibits anti-symmetry, some portion of the between-sides variation may have a genetic basis; hence, the between-sides variance may not purely be a product of developmental noise [28]. Skewness and kurtosis statistics were used to describe any departure from normality.

#### Fluctuating asymmetry (FA) of Dermatoglyphics

*Qualitative FA* Fingerprint patterns are not morphometric traits that have a readily measurable size and shape. However, pattern types tend to be identical on homologous fingers. Hence, the degree of pattern discordance can be used as a measure of fluctuating asymmetry [29]. Therefore, the bilateral asymmetries of the finger print patterns were assessed using pattern discordance [17, 29, 30]. For example, if the R and L thumb of a person have the same pattern such as an UL on both thumbs, then the patterns are concordant. If the digital patterns differ, for instance, an UL on the right thumb and a PW on the left thumb, then the patterns are discordant.

*Quantitative FA* Based on standard recommendations, we used two methods to calculate fluctuating asymmetry [18]. These two methods were used by Saha et al. [31] during their study on dermatoglyphic asymmetry in psychosis.

##### FA calculated using correlation method

The comparisons were made in higher ridge counts between each homologous fingers of the R and L hands using Pearson product-moment correlation coefficients (*r*) [32]. The difference in *r* value of ridge count between cases and controls was calculated using Fisher’s z-transformation [31]. The square of the *r* of the two variables is a measure of their common variance and 1-*r*^2^ is an estimate of error variance (coefficient of indetermination) [31, 33] This is an estimate of their unshared variance and therefore of fluctuating asymmetry [29]. This method is not affected by DA [18].

##### Adjusted FA difference method

FA was obtained by subtracting the L hand ridge count from the R hand count of each homolog finger and dividing by the average of the sum of ridge count in both hand, and then taking the absolute value of the quotient [34]. The presence of DA artificially inflates the values of the FA difference scores. Therefore, if a significant DA was identified, FA difference scores for all measures were adjusted by subtracting the average mean R minus L difference (mean (R-L)/2) from the side with the larger mean and adding it to the smaller side of all individuals in the sample [35].

### Reliability of observation

Intra-observer reliability for pattern classification was assessed by comparing observations made by the same observer during initial and later evaluations. Thumbprint patterns of 50 individuals were initially examined and recorded by the principle investigator. The same 50 individuals’ patterns were shuffled, and the thumbprints were examined and recorded a second time one month later by the principle investigator (PI). Similarly, palm print patterns were scored on two occasions. PI was blinded to his previous observation. Intra-observer reliability agreement for the pattern type on each finger was assessed with Cohen’s kappa (κ) coefficient of agreement. Interpretation of κ was based on the criteria of Landis and Koch [36].

Intra-rater reliability was also assessed by comparing two ridge counting made by the same rater during the first and second evaluations. The D1 ridge counts of 50 individuals were initially recorded by the principle investigator. These 50 individuals’ patterns were then shuffled, and their ridge counts were recorded a second time 1 month apart by the principle investigator. A similar procedure was used for palmar A-B RC. The reliability analysis was assessed by calculating the intraclass correlation coefficient (ICC) for the two recordings [37].

## Results

### Sample size and composition

A total of 180 (90 females and 90 males) CKDu cases were selected. The mean age of females was 60 years (SD = 10), and the mean age of males was 61 years (SD = 10). The EC consisted of 180 (90 females and 90 males) participants. The mean age of females was 44 years (SD = 10), and the mean age of males was 44 years (SD = 11). The NEC consisted of 180 (90 females and 90 males) participants; the mean age of females was 46 years (SD = 14) and mean age of males was 48 years (SD = 14).

### Reliability of the dermatoglyphics analysis

There was almost perfect agreement between the two observations in both digital dermatoglyphics (*κ* = 0.932 [95% CI, 0.84 to 1.02], *p* < .0005) and palmar dermatoglyphic patterns (*κ* = 0.912 [95% CI, 0.82 to 1.01], *p* < .0001). A nearly exact agreement also was observed between the two observations on ridge counting of D1 (ICC = 0.995 [95% CI, 0.992 to 0.997], *p* < .0001) and A-B RC on R palm (ICC = 0.997 [95% CI, 0.994 to 0.998], *p* < .0001).

### Qualitative dermatoglyphics

In the following tables, results are presented with three probability values according to two-way tests between CKDu cases and EC, between CKDu cases and NEC, and also between EC and NEC.

### Digital dermatoglyphics of males—Additional file 1: Table S1

There were no statistically significant differences in frequencies of digital patterns observed in the R and L hands between CKDu cases and control groups (EC and NEC). However, PW frequency was significantly higher on L D1 in EC compared to NEC.

### Digital dermatoglyphics of females—Additional file 2: Table S2

There was a significantly higher percentage of UL pattern observed on L D5 in females between CKDu patients, while PW frequency was significantly higher on L D1 of the EC compared to CKDu cases.

When comparing CKDu cases with NEC, a significantly higher percentage of DL was observed on L D3 while a higher percentage of CPL was seen on L D2. The percentage of PW was significantly lower on L D2. In contrast, a significantly lower percentage of PA was observed on R D3 in CKDu cases.

A comparison of EC and NEC showed a significantly lower percentage of PA in EC on R D3, while a significantly higher PW frequency was found on L D4 of EC.

### Palmar dermatoglyphics of males—Additional file 3: Table S3 and Additional file 4: Table S4

No significant differences between cases and controls were found regarding male frequencies of palmar loop patterns.

Regarding triradii, a significantly higher percentage of triradii a^1^ on the R hand was observed in both control groups and cases.

Conversely, there is a significantly higher percentage of triradii t on the L hand of cases versus NEC. However, there was also a significantly higher frequency on the L hand triradii t in EC when compared with the other control group NEC.

### Palmar dermatoglyphics of females—Additional file 5: Table S5 and Additional file 6: Table S6

For the palm, a comparison of CKDu cases with NEC yielded these results; the palmar loop IIIT was found to be significantly more common on the R hand of cases. For control groups, there were three palmar patterns (II and H on L hand, and IV on R hand) in which EC consistently showed significantly higher loop frequencies than NEC.

With regard to triradii, Triradii a^1^ was significantly less frequent in cases against both EC and NEC on the R hand while triradii t^11^ was significantly more frequent in cases versus NEC for both hands. Triradius c^1^ showed a mixed result, CKDu cases had a significantly lower c^1^ frequency than EC for both hands, but EC also had a significantly higher c^1^ frequency than NEC for the L hand.

### FA of patterns—Table 1

Fluctuating asymmetry of pattern discordance was found to be significant on D3 among males and on D2 among females when comparing CKDu cases with NEC.

**Table 1:** Fluctuating asymmetry of pattern discordance

		C		EC		C Vs EC P	NEC		C Vs NEC P	EC Vs NEC P
		Pattern Discordant		Pattern Discordant			Pattern Discordant			
		%	N	%	N		%	N		
Male	D1	31.11	28	35.56	32	0.53	31.11	28	1.00	0.53
	D2	45.56	41	45.56	41	1.00	50.00	45	0.55	0.55
	D3	34.44	31	27.78	25	0.33	18.89	17	0.02*	0.16
	D4	30.00	27	28.89	26	0.87	34.44	31	0.52	0.42
	D5	21.11	19	17.78	16	0.57	25.56	23	0.48	0.21
Female	D1	33.33	30	42.22	38	0.22	31.11	28	0.75	0.12
	D2	57.78	52	46.67	42	0.14	33.33	30	0.00*	0.07
	D3	26.67	24	26.67	24	1.00	28.89	26	1.00	0.74
	D4	26.67	24	30.00	27	0.62	28.89	26	0.74	0.87
	D5	16.67	15	26.67	24	0.10	17.78	16	0.84	0.15

*D* digit, *C* cases, *EC* endemic control, *NEC* non endemic control, *N* number of values, *P* P value, * significant values

### Quantitative dermatoglyphics—Table 2

General results of quantitative variables of the between cases and control groups descriptive statistics and significance tests are shown in Table 2. As can be seen, only A-B RC was found to be significantly different in the paired case control tests, in which CKDu cases for both sexes had higher values, or higher palmar ridge counts, than EC, as well as for NEC females, and marginally non-significant in males. There was also a fairly consistent tendency for CKDu cases to have higher PII values along with larger finger ridge counts than control groups. This would weakly signal more complex patterns developed in CKDu patients.

**Table 2:** Quantitative variables of cases and controls

		Cases		Endemic control		Non-endemic control	
		Mean	SD	Mean	SD	Mean	SD
PII	Male (*N* = 90)	13.68	3.28	12.34	3.66	13.32	3.82
	Female (*N* = 90)	13.08	3.29	12.48	3.12	13.16	3.71
TRC	Male (*N* = 90)	151.62	41.82	144.02	42.78	149.63	47.57
	Female (*N* = 90)	143.32	44.12	146.87	41.31	143.84	51.07
A-B RC	Male (*N* = 90)	83.81	10.59	76.99^*^	10.38	81.82	11.08
	Female (*N* = 90)	85.10	12.36	77.67^*^	8.93	81.27^§^	10.26

*PII* pattern intensity index, *TRC* total ridge count, *A-B RC* A-B ridge count, *N* number of values, *SD* standard deviation

*Mann-Whitney test *P* < 0.001

^§^Mann-Whitney test *P* < 0.02

### Asymmetry of pattern distributions—test for normality

Since platykurtosis was not present in the distributions of right to left ridge count differences, the occurrence of anti-symmetry is unlikely. Thus, there is some support for interpreting FA in terms of environmental effect rather than due to genetic effect.

### Directional asymmetry—Additional file 7: Table S7

Significant DA findings were observed for females on D1RC in both EC and NEC groups, and also on D1 RC in males of CKDu cases.

### Fluctuating asymmetry—difference method Table 3

Significant differences in FA were observed for D2 RC in cases versus both control groups in females. In females, TRC was also significant in the cases versus EC test, along with that for A-B RC for the cases versus NEC test. For males, significant differences can be seen with regard to CKDu cases for A-B RC and the EC group, and for D3 RC, TRC, and A-B RC in the tests against the NEC control group. These results signal a moderate degree for distinguishing between cases and controls. However, significant differences also were found for six variables in tests between EC and NEC control groups, namely, for D5 RC and TRC in females and D3 RC, D4 RC, TRC, and A-B RC in males. Consequently, there appears to be nearly an even mix of FA differences between CKDu and control groups, that is, for the FA difference method, CKDu shows greater FA values in half of the significant test results, while the control groups themselves show higher FA in the remaining half.

**Table 3:** Fluctuating asymmetry—difference method

		Cases		EC		C vs EC P	NEC		C vs NEC P	EC vs NEC P
		Mean	SD	Mean	SD		Mean	SD		
Female	D1 RC	0.24	0.21	0.19	0.15	0.10	0.18	0.17	0.07	0.74
	D2 RC	0.36	0.34	0.24	0.27	0.01*	0.27	0.22	0.03*	0.5
	D3 RC	0.23	0.23	0.22	0.20	0.74	0.22	0.20	0.65	0.89
	D4 RC	0.15	0.14	0.15	0.12	0.88	0.18	0.17	0.19	0.13
	D5 RC	0.18	0.14	0.15	0.12	0.08	0.20	0.17	0.34	0.01*
	TRC	0.10	0.08	0.08	0.07	0.04*	0.11	0.09	0.73	0.02*
	A-B RC	0.11	0.07	0.09	0.06	0.10	0.08	0.06	0.01*	0.27
Male	D1 RC	0.18	0.18	0.18	0.17	0.89	0.22	0.2	0.22	0.16
	D2 RC	0.26	0.24	0.25	0.27	0.75	0.3	0.33	0.42	0.30
	D3 RC	0.23	0.21	0.24	0.27	0.90	0.17	0.16	0.02*	0.04*
	D4 RC	0.15	0.12	0.18	0.17	0.22	0.13	0.11	0.28	0.04*
	D5 RC	0.21	0.19	0.17	0.16	0.16	0.16	0.15	0.11	0.83
	TRC	0.10	0.07	0.10	0.12	0.78	0.47	0.42	0.00*	0.00*
	A-B RC	0.07	0.06	0.11	0.16	0.04*	0.41	0.17	0.00*	0.00*

*TRC* finger ridge count, *A-B RC* A-B ridge count

*Significant difference (F-test based on general linear model with adjusted probability estimates with Tukey-Kramer test)

### Fluctuating asymmetry correlation method—Table 4

The FA-correlation method only identified significant differences of FA in D3 RC and D4 RC between cases and NEC in males, and in D2 RC in females. Males alone accounted for all of the significant FA-correlation tests of EC versus NEC control groups for the three variables of D3 RC, D4 RC, and A-B RC.

**Table 4:** Fluctuating asymmetry—correlation method

		CASES	EC	CASES vs EC P	NEC	CASES vs NEC P	EC vs NEC P
		1-*r*^2^	1-*r*^2^		1-*r*^2^		
Female	D1 RC	0.46	0.47	0.93	0.42	0.68	0.62
	D2 RC	0.61	0.47	0.22	0.38	0.03*	0.36
	D3 RC	0.46	0.42	0.71	0.37	0.31	0.52
	D4 RC	0.33	0.33	0.92	0.38	0.52	0.58
	D5 RC	0.39	0.30	0.30	0.39	0.98	0.31
	TRC	0.17	0.18	0.85	0.15	0.65	0.52
	A-B RC	0.46	0.49	0.73	0.34	0.23	0.12
Male	D1 RC	0.42	0.38	0.65	0.48	0.60	0.32
	D2 RC	0.47	0.47	0.95	0.55	0.45	0.22
	D3 RC	0.48	0.59	0.31	0.28	0.02*	0.00*
	D4 RC	0.39	0.41	0.80	0.21	0.02*	0.01*
	D5 RC	0.59	0.44	0.19	0.41	0.10	0.73
	TRC	0.18	0.17	0.76	0.17	0.88	0.87
	A-B RC	0.38	0.55	0.10	0.32	0.45	0.02*

*TRC* total ridge count, *A-B RC* A-B ridge count, *r* pearson product-moment correlation coefficients, *P* P value, * significant values

*Fisher’s Z test for significant differences

### The summary of positive findings—Table 5

There were many significant dermatoglyphic variables observed between cases and NEC as well as between EC and NEC. Importantly, significant FA results of the qualitative variables were only observed between cases and NEC. There were significant FA (FA correlation method) results observed in D3 and D4 of the males between cases and NEC, and also between EC and NEC. Similarly, significant FA (FA-adjusted method) results were observed in D3, TRC, and ABR of males between cases and NEC, as well as between EC and NEC.

**Table 5:** Summary of positive findings

Dermatoglyphic variable	Cases vs EC	Cases vs NEC	EC vs NEC
Qualitative Digital dermatoglyphics			
Male			LD1 more PW in EC
Female	LD5 more UL in cases LD1 less PW in cases	LD2 more PW in NEC LD3 more DL in cases LD2 more CPL in cases RD3 less PA in cases	LD4 more PW in EC RD3 less PA in EC
Qualitative Palmar dermatoglyphics			
Male	Less a^1^ in RH of cases	Less a^1^ in RH of cases More III^T^ in RH of the cases	More II in RH EC More Ĥ in RH of EC More IV LH of EC
Female	More a^1^ in RH of EC More c^1^ in LH of EC	More t in LH of cases More a^1^ in RH of NEC More t^11^ in RH of cases More t^11^ in LH of cases	More t in LH of EC More c^1^ in LH of EC
Qualitative FA			
Male		D3	
Female		D2	
Quantitative FA correlation			
Male		D3 D4	D3 D4 A-B RC
Female		D2	
FA adjusted			
Male	A-B RC	A-B RC D3 TRC	A-B RC D3 D4 TRC
Female	TRC D2	A-B RC D2	D5 TRC

*EC* endemic control, *NEC* non-endemic control, *LD* left side digit, *RD* right side digit, *D* digit, *LH* left hand, *RH* right hand, *UL* ulnar loop, *PW* plain whorl, *DL* double loop, *CPL* central pocket loop, *PA* plain arch, *TRC* total ridge count, *A-B RC* A-B ridge count

## Discussion

This study showed that several qualitative dermatoglyphic variables had significant association with CKDu. Also, the FA of pattern discordance (R vs L hands) between CKDu cases and control group were significant in several digits. It is of interest to note that the overall digital dermatoglyphic pattern frequencies in each of the three groups were in line with those of the Sinhalese population [27]. There were few previous studies that provided evidence to support an association between qualitative dermatoglyphics and kidney diseases [7].

In our study, the FA of the ridge count was found to be significant in several digits as well as for A-B RC and TRC. Here is our basis for interpreting these findings. The development of dermal ridge patterns is controlled by the process of initial appearance and later regression of volar pads [6]. The development of ridges occurs at the dermal-epidermal junction [38]. The formation of volar pads first appear on the fingertips on the sixth to seventh week of fetal development and are prominent over subsequent weeks [6, 38]. Volar pads start to diminish from the fifth month and disappear by the sixth month [6, 38]. According to Bonnevie [6, 39] the position and size of the volar pad is responsible for pattern configuration. Thus, a small, low pad forms arch patterns, while an elevated, large symmetrical-shaped pad forms whorls, and asymmetrical pads form loops.

Importantly, unknown environmental factors could be a common cause for exerting stress on the development of both ridge patterns and kidneys. However the differences, there were few previous studies that provided evidence to support an association between qualitative dermatoglyphics and kidney diseases [7]. In our study, the FA of the ridge count was found to be significant in several digits as well as for A-B RC and TRC. Here is our basis for interpreting these findings. The development of dermal ridge patterns is controlled by the process of initial appearance and later regression of volar pads [6]. The development of ridges occurs at the dermal-epidermal junction [40]. The formation of volar pads first appear on the fingertips on the sixth to seventh week of fetal development and are prominent over subsequent weeks [6, 40]. Volar pads start to diminish from the fifth month and disappear by the sixth month [6, 40]. According to Bonnevie [6, 38], the position and size of the volar pad are responsible for pattern configuration. Thus, a small, low pad forms arch patterns, while an elevated, large symmetrical-shaped pad forms whorls, and asymmetrical pads form loops.

Importantly, unknown environmental factors could be a common cause for exerting stress on the development of both ridge patterns and kidneys. However, the difference for TRC in CKDu cases is only observed between EC female and NEC males. TRC has been shown to be less influenced by developmental insult arising from environmental factors [12].

The triradii a^1^ variable was less evident in CKDu cases in both genders when compared to both control groups. Among males who lived in the endemic region, FA of A-B RC was found to be significant. Both of these variables occur in interdigital area two (ID 2). Triradii a^1^ is an accessory pattern that appears in the vicinity of triradii A, while A-B RC is a measure of the size of the ID 2 [11]. With regard to developmental timing, the fetal pad for the ID 2 area appears first followed by the pads on fingers, and palmar ridges form earlier and develop over a longer period than digital ridges [39, 41]. The A-B RC asymmetry is reported to be ideal for indexing developmental canalization [41, 42]. The A-B RC could be highly sensitive to environmental noises, and these results in poor canalization in CKDu cases who are exposed to unknown environmental factors during development. Environmental stressors during the second-trimester were proposed to underlie an association between familial schizophrenia and dermatoglyphics [43]. Similarly, the buffering capacity for environmental stress could be lessened in CKDu patients. Therefore, these dermatoglyphic variables could be useful in the early detection of communities who are at risk of developing CKDu.

Could certain environmental factors simultaneously affect the development of CKDu and dermatoglyphics in the North Central Province? Environment has been convincingly shown to have had an impact on the development of dermatoglyphics during the prenatal period [13]. Furthermore, several environmental factors were found to be significantly associated with CKDu patients such as pesticides, heavy metals exposure, and contaminated drinking water [4]. Quite possibly, fetal exposure to these identified agents or as yet unknown factors could alter the development of both dermatoglyphics and kidneys that make them more vulnerable to disease later in life.

A study by Kahn et al. [44] showed L and R hand RC differences between D1 and D5 in the offspring of mothers who were exposed to hunger during gestation. Further, they have showed that the same dermatoglyphic variables were significantly associated with diabetes in offspring who were exposed to environmental stress during their gestation [23]. Related to kidney development, maternal dietary imbalance was reported to be a cause of an increased risk for insufficient renal function [45]. These studies clearly indicate the potential application of dermatoglyphic markers as disease risk factors.

Previous studies have reported significant DA in thumbs [31, 46], and we assessed DA for all quantitative variables. We used adjusted FA difference that overcame the confounding effect of DA [18]. This would make FA a suitable variable for investigating possible environmental effects on dermatoglyphic development. Furthermore, we were able to recruit participants who belonged to the Sinhalese ethnic group who were identified as Sinhalese for at least two generations without miscegenation. The analyses of prints were done in sex-wise subcategories thereby removing any sex-related confounders. Measurement error could have been a limitation of this study, although it was minimized because prints were examined by a single rater under strict supervision.

Despite our careful sampling procedure and methodology considerations, there were several variables found to be statistically significant between the control groups, EC, and NEC. We did exclude the presence of CKD in those individuals in EC through both clinical and laboratory investigations. However, the overall prevalence of CKDu in Anuradhapura is 15.1%. Therefore, there could be individuals in the EC group who could have been at risk for developing CKDu in the future. Therefore, our control group from EC might not have been an ideal complement in testing dermatoglyphic variables with the other control group from NEC. In essence, since our study provides evidence that dermatoglyphics of some individuals in EC could have been altered similar to that of CKDu cases, there is a possibility that fetal developmental insult from unknown underlying causative factors of CKDu also occurred in some individuals in the EC group.

In summing up this report, some measures of digital and palmar pattern variation, and also some of the derived measures of directional and fluctuating asymmetry, were found to be significant. What then, is the application for these results? Renal damage was evident among children living in CKDu endemic region [47]; therefore, it is vital to develop methods to identify those individuals and populations who are at risk of developing CKD later in life. Furthermore, methods should be cost effective and easy to administer. Since dermatoglyphics fully develop in early fetal life and thereafter remain unchanged, these variables can be used to identify individual children or a group of children who might beat greater risk of developing CKD in their future.

When dermatoglyphic findings are combined with clinical features and results of other investigations, they can strengthen a risk of disease diagnosis so that preventive methods can commence at an early age. The photographic method for taking dermatoglyphic prints can be conducted as a point-of-care appointment, where it would be of low cost, rapid, and non-invasive, and thus appropriate for use in low socioeconomic countries that could have less advanced diagnostic facilities. This method is so convenient and practical that it could be done during the neonatal period on a routine basis. Due to rapid advancement in technology, high-quality cameras are now available in mobile phones that could be used even in busy clinics.

Future studies should be directed toward developing methods to predict future occurrence of CKDu in risk populations. Dermatoglyphic variables such as and A-B RC found in ID 2 were significant in CKDu patients. By following similar methods described by Acree [48], it is possible to identify ridge density in a predefined square in ID 2 in CKDu group, and then the probabilities of occurrence of CKDu for a given dermal ridge count could be based on Bayesian inference.

In conclusion, to some extent, dermatoglyphics of CKDu patients in this study were altered compared to non-diseased individuals. Therefore, they may be used as an easily accessible tool to assist in the early detection of groups of people who are at increased risk for developing CKDu. Of course, further studies with larger sample sizes are needed to verify and extend our findings so that eventually highly reliable dermatoglyphic biomarkers may be discovered.

## Supplementary information

**Additional file 1: Table S1.** Digital dermatoglyphics of males.

**Additional file 2: Table S2.** Digital dermatoglyphics of females.

**Additional file 3: Table S3.** Palmar dermatoglyphics (loops) of males.

**Additional file 4: Table S4.** Palmar dermatoglyphics (triradii) of males.

**Additional file 5: Table S5.** Palmar dermatoglyphics (loops) of females.

**Additional file 6: Table S6.** Palmar dermatoglyphics (triradii) of females.

**Additional file 7: Table S7.** Directional asymmetry.

## Data Availability

Please contact the corresponding author for data requests.

## Abbreviations

A: Accidental whorl
A-B RC: A-B ridge count
ACR: Albumin to creatinine ratio
CINAC: Chronic interstitial nephritis in agricultural communities
CKD: Chronic kidney disease
CKDu: Chronic kidney disease of unknown etiology
CPL: Central pocket loop
DA: Directional asymmetry
DL: Double loop
DS: Divisional secretariat
EC: Endemic control
eGFR: Estimated glomerular filtration rate
FA: Fluctuating asymmetry
HbA1c: Hemoglobin A1 C
ICC: Intraclass correlation coefficient
ID: Inter digital
IS: Image stabilizer
LH: Left hand
NCP: North central province
NEC: Non-endemic control
OR: Odds ratio
PA: Plain arc
PII: Pattern intensity index
PW: Plain whorl
RH: Right hand
RL: Radial loop
SD: Standard deviation
STM: Stepper Motor
TA: Tented arch
TIFF: Tagged Image File Format
TRC: Total ridge count
UL: Ulnar loop
