# Supplementary material for: Qualitative and quantitative dermatoglyphics of chronic kidney disease of unknown origin (CKDu) in Sri Lanka
Source: J Physiol Anthropol. 2020 Jan 17;39:1. doi: 10.1186/s40101-019-0207-0 (PMC6967092; doi:10.1186/s40101-019-0207-0)
Supplement: Supplementary file 1 — Additional file 1: Table S1. Digital dermatoglyphics of males. [file 40101_2019_207_MOESM1_ESM.docx]

| **Table S1** Digital dermatoglyphics of males | | | | | | | | | | | | | | | | | | | | | | | | | | | | |
| --- | --- | --- | --- | --- | --- | --- | --- | --- | --- | --- | --- | --- | --- | --- | --- | --- | --- | --- | --- | --- | --- | --- | --- | --- | --- | --- | --- | --- |
|  | | Ulnar loop | | | Radial loop | | | Plain whorl | | | Double loop | | | Central pocket loop | | | Accidental | | | Plain arch | | | Tented arch | | | Unclassified | | |
|  |  | N | % | P | N | % | P | N | % | P | N | % | P | N | % | P | N | % | P | N | % | P | N | % | P | N | % | P |
| R D1 | Cases | 45 | 51.1 |  | 0 | 0 | 1 | 27 | 30.7 |  | 14 | 15.9 |  | 0 | 0 |  | 0 | 0 |  | 2 | 2.3 |  | 0 | 0 |  | 0 | 0 |  |
|  | EC | 43 | 48.9 | 0.77 | 1 | 1.1 |  | 32 | 36.4 | 0.42 | 8 | 9.1 | 0.17 | 0 | 0 | 1 | 0 | 0 | 1 | 4 | 4.5 | 0.68 | 0 | 0 | 1 | 0 | 0 | 1 |
|  | NEC | 48 | 53.9 | 0.71 | 0 | 0 | 1 | 27 | 30.3 | 0.96 | 11 | 12.4 | 0.5 | 0 | 0 | 1 | 0 | 0 | 1 | 1 | 1.1 | 0.62 | 0 | 0 | 1 | 2 | 2.2 | 0.5 |
|  | EC VS NEC |  |  | 0.5 |  |  | 0.5 |  |  | 0.4 |  |  | 0.48 |  |  | 1 |  |  | 1 |  |  | 0.21 |  |  | 1 |  |  | 0.5 |
| R D2 | Cases | 38 | 42.2 |  | 6 | 6.7 |  | 31 | 34.4 |  | 7 | 7.8 |  | 1 | 1.1 |  | 2 | 0 |  | 3 | 3.3 |  | 1 | 1.1 |  | 1 | 1.1 |  |
|  | EC | 36 | 40.4 | 0.81 | 9 | 10.1 | 0.41 | 32 | 36 | 0.83 | 3 | 3.4 | 0.2 | 1 | 1.1 | 1 | 1 | 0 | 0.57 | 4 | 4.5 | 0.99 | 2 | 2.2 | 0.55 | 1 | 1.1 | 0.99 |
|  | NEC | 36 | 40.9 | 0.86 | 5 | 5.7 | 0.79 | 28 | 31.8 | 0.83 | 8 | 9.1 | 0.75 | 3 | 3.4 | 0.37 | 1 | 1.1 | 1 | 4 | 4.5 | 0.72 | 3 | 3.4 | 0.37 | 0 | 0 | 0.99 |
|  | EC VS NEC |  |  | 0.95 |  |  | 0.4 |  |  | 0.56 |  |  | 0.13 |  |  | 0.37 |  |  | 1 |  |  | 1 |  |  | 0.68 |  |  | 1 |
| R D3 | Cases | 69 | 76.7 |  | 0 | 0 |  | 17 | 18.9 |  | 2 | 2.2 |  | 1 | 1.1 |  | 0 | 0 |  | 1 | 1.1 |  | 0 | 0 |  | 0 | 0 |  |
|  | EC | 62 | 69.7 | 0.29 | 1 | 1.1 | 0.31 | 19 | 21.3 | 0.68 | 1 | 1.1 | 1 | 3 | 3.4 | 0.37 | 0 | 0 | 1 | 2 | 2.2 | 0.62 | 0 | 0 | 1 | 1 | 1.1 | 0.5 |
|  | NEC | 66 | 73.3 | 0.61 | 0 | 0 | 1 | 19 | 21.1 | 0.68 | 2 | 2.2 | 1 | 1 | 1.1 | 1 | 0 | 0 | 1 | 2 | 2.2 | 1 | 0 | 0 | 1 | 0 | 0 | 1 |
|  | EC VS NEC |  |  | 0.59 |  |  | 0.5 |  |  | 0.97 |  |  | 1 |  |  | 0.37 |  |  | 1 |  |  | 1 |  |  | 1 |  |  | 1 |
| R D4 | Cases | 29 | 32.6 |  | 2 | 2.2 |  | 47 | 52.8 |  | 1 | 1.1 |  | 9 | 10.1 |  | 0 | 0 | 1 | 0 | 0 |  | 1 | 1.1 |  | 0 | 0 |  |
|  | EC | 33 | 37.1 | 0.53 | 0 | 0 | 0.5 | 48 | 53.9 | 0.88 | 0 | 0 | 1 | 5 | 5.6 | 0.4 | 0 | 0 | 1 | 2 | 2.2 | 0.5 | 1 | 1.1 | 1 | 0 | 0 | 1 |
|  | NEC | 32 | 35.6 | 0.68 | 1 | 1.1 | 0.62 | 44 | 48.9 | 0.89 | 1 | 1.1 | 1 | 10 | 11.1 | 0.83 | 1 | 1.1 | 1 | 1 | 1.1 | 1 | 0 | 0 | 1 | 0 | 0 | 1 |
|  | EC VS NEC |  |  | 0.83 |  |  | 1 |  |  | 0.5 |  |  | 1 |  |  | 0.28 |  |  | 1 |  |  | 0.62 |  |  | 1 |  |  | 1 |
| R D5 | Cases | 70 | 77.8 |  | 0 | 0 |  | 17 | 18.9 |  | 1 | 1.1 |  | 2 | 2.2 |  | 0 | 0 |  | 0 | 0 |  | 0 | 0 |  | 0 | 0 |  |
|  | EC | 72 | 80 | 0.71 | 0 | 0 | 1 | 15 | 16.7 | 0.7 | 1 | 1.1 | 1 | 2 | 2.2 | 1 | 0 | 0 | 1 | 0 | 0 | 1 | 0 | 0 | 1 | 0 | 0 | 1 |
|  | NEC | 65 | 72.2 | 0.39 | 0 | 0 | 1 | 17 | 18.9 | 0.94 | 0 | 0 | 1 | 8 | 8.9 | 0.1 | 0 | 0 | 1 | 0 | 0 | 1 | 0 | 0 | 1 | 0 | 0 | 1 |
|  | EC VS NEC |  |  | 0.22 |  |  | 1 |  |  | 0.7 |  |  | 1 |  |  | 0.1 |  |  | 1 |  |  | 1 |  |  | 1 |  |  | 1 |
| L D1 | Cases | 43 | 48.9 |  | 0 | 0 |  | 23 | 26.1 |  | 19 | 21.6 |  | 0 | 0 |  | 1 | 0 |  | 2 | 2.3 |  | 0 | 0 |  | 0 | 0 |  |
|  | EC | 41 | 46.6 | 0.71 | 0 | 0 | 1 | 30 | 34.1 | 0.27 | 11 | 12.4 | 0.1 | 1 | 1.1 | 1 | 2 | 0 | 1 | 4 | 4.5 | 0.68 | 0 | 0 | 1 | 0 | 0 | 1 |
|  | NEC | 49 | 55.7 | 0.37 | 0 | 0 | 1 | 15 | 17 | 0.14 | 20 | 22.7 | 0.86 | 0 | 0 | 1 | 1 | 1.1 | 1 | 3 | 3.4 | 1 | 0 | 0 | 1 | 0 | 0 | 1 |
|  | EC VS NEC |  |  | 0.23 |  |  | 1 |  |  | 0.01* |  |  | 0.07 |  |  | 1 |  |  | 1 |  |  | 1 |  |  | 1 |  |  | 1 |
| L D2 | Cases | 37 | 41.1 | . | 7 | 7.8 |  | 30 | 33.3 |  | 8 | 8.9 |  | 1 | 1.1 |  | 0 | 0 |  | 4 | 4.4 |  | 3 | 3.3 |  | 0 | 0 |  |
|  | EC | 39 | 43.8 | 0.71 | 9 | 10.1 | 0.58 | 26 | 29.2 | 0.55 | 5 | 5.6 | 0.57 | 0 | 0 | 1 | 0 | 0 | 1 | 8 | 9 | 0.25 | 2 | 2.2 | 1 | 0 | 0 | 1 |
|  | NEC | 34 | 38.2 | 0.69 | 8 | 9 | 0.77 | 26 | 29.2 | 0.55 | 9 | 10.1 | 0.78 | 0 | 0 | 1 | 3 | 3.4 | 0.12 | 5 | 5.6 | 0.75 | 3 | 3.4 | 1 | 1 | 1.1 | 1 |
|  | EC VS NEC |  |  | 0.45 |  |  | 0.8 |  |  | 1 |  |  | 0.4 |  |  | 1 |  |  | 0.25 |  |  | 0.57 |  |  | 1 |  |  | 1 |
| L D3 | Cases | 57 | 63.3 |  | 2 | 2.2 |  | 16 | 17.8 |  | 6 | 6.7 |  | 1 | 1.1 |  | 0 | 0 |  | 5 | 5.6 |  | 3 | 3.3 |  | 0 | 0 |  |
|  | EC | 58 | 65.2 | 0.8 | 1 | 1.1 | 1 | 18 | 20.2 | 0.68 | 5 | 5.6 | 1 | 0 | 0 | 1 | 0 | 0 | 1 | 7 | 7.9 | 0.57 | 0 | 0 | 0.25 | 0 | 0 | 1 |
|  | NEC | 60 | 66.7 | 0.64 | 1 | 1.1 | 1 | 20 | 22.2 | 0.46 | 2 | 2.2 | 0.28 | 1 | 1.1 | 1 | 0 | 0 | 1 | 5 | 5.6 | 1 | 1 | 1.1 | 0.62 | 0 | 0 | 1 |
|  | EC VS NEC |  |  | 0.83 |  |  | 1 |  |  | 0.74 |  |  | 0.28 |  |  | 1 |  |  | 1 |  |  | 0.57 |  |  | 1 |  |  | 1 |
| L D4 | Cases | 33 | 37.1 |  | 0 | 0 |  | 45 | 50.6 |  | 2 | 2.2 |  | 10 | 11.1 |  | 0 | 0 |  | 0 | 0 |  | 0 | 0 |  | 0 | 0 |  |
|  | EC | 34 | 38.2 | 0.83 | 1 | 1.1 | 0.5 | 43 | 48.3 | 0.82 | 3 | 3.4 | 0.68 | 6 | 6.7 | 0.31 | 0 | 0 | 1 | 2 | 2.2 | 0.25 | 0 | 0 | 1 | 0 | 0 | 1 |
|  | NEC | 44 | 48.9 | 0.1 | 0 | 0 | 1 | 35 | 38.9 | 0.13 | 3 | 3.3 | 1 | 6 | 6.7 | 0.29 | 0 | 0 | 1 | 2 | 2.2 | 0.5 | 0 | 0 | 1 | 0 | 0 | 1 |
|  | EC VS NEC |  |  | 0.15 |  |  | 1 |  |  | 0.2 |  |  | 1 |  |  | 1 |  |  | 1 |  |  | 1 |  |  | 1 |  |  | 1 |
| L D5 | Cases | 68 | 75.6 |  | 1 | 1.1 |  | 15 | 16.7 |  | 1 | 1.1 |  | 5 | 5.6 |  | 0 | 0 |  | 0 | 0 |  | 0 | 0 |  | 0 | 0 |  |
|  | EC | 71 | 78.9 | 0.59 | 0 | 0 | 1 | 15 | 16.7 | 0.68 | 1 | 1.1 | 1 | 2 | 2.2 | 0.72 | 0 | 0 | 1 | 1 | 1.1 | 1 | 0 | 0 | 1 | 0 | 0 | 1 |
|  | NEC | 68 | 76.4 | 0.89 | 0 | 0 | 1 | 11 | 12.4 | 0.41 | 3 | 3.4 | 0.37 | 6 | 6.7 | 0.74 | 0 | 0 | 1 | 1 | 1.1 | 1 | 0 | 0 | 1 | 0 | 0 | 1 |
|  | EC VS NEC |  |  | 0.69 |  |  | 1 |  |  | 0.68 |  |  | 0.68 |  |  | 0.33 |  |  | 1 |  |  | 1 |  |  | 1 |  |  | 1 |
| *D digit, R* right, *L* left, *EC* endemic control, *NEC* non endemic control, *N* number of values, *** significant values | | | | | | | | | | | | | | | | | | | | | | | | | | | | |
