# Supplementary material for: Qualitative and quantitative dermatoglyphics of chronic kidney disease of unknown origin (CKDu) in Sri Lanka
Source: J Physiol Anthropol. 2020 Jan 17;39:1. doi: 10.1186/s40101-019-0207-0 (PMC6967092; doi:10.1186/s40101-019-0207-0)
Supplement: Supplementary file 2 — Additional file 2: Table S2. Digital dermatoglyphics of females. [file 40101_2019_207_MOESM2_ESM.docx]

| **Table S2** Digital dermatoglyphics of females | | | | | | | | | | | | | | | | | | | | | | | | | | | | |
| --- | --- | --- | --- | --- | --- | --- | --- | --- | --- | --- | --- | --- | --- | --- | --- | --- | --- | --- | --- | --- | --- | --- | --- | --- | --- | --- | --- | --- |
|  | | Ulnar loop | | | Radial loop | | | Plain whorl | | | Double loop | | | Central pocket loop | | | Accidental | | | Plain arch | | | Tented arch | | | Unclassified | | |
|  |  | N | % | P | N | % | P | N | % | P | N | % | P | N | % | P | N | % | P | N | % | P | N | % | P | N | % | P |
| RD1 | Cases | 40 | 44.4 |  | 0 | 0 |  | 24 | 26.7 |  | 18 | 20 |  | 0 | 0 |  | 0 | 0 |  | 8 | 8.9 |  | 0 | 0 |  | 0 | 0 |  |
|  | EC | 49 | 54.4 | 0.18 | 0 | 0 | 1 | 27 | 30 | 0.62 | 12 | 13.3 | 0.23 | 0 | 0 | 1 | 0 | 0 | 1 | 2 | 2.2 | 0.1 | 2 | 2.2 | 1 | 0 | 0 | 1 |
|  | NEC | 52 | 57.8 | 0.07 | 0 | 0 | 1 | 22 | 24.4 | 0.73 | 13 | 14.4 | 0.32 | 1 | 1.1 | 1 | 0 | 0 | 1 | 2 | 2.2 | 0.1 | 0 | 0 | 1 | 0 | 0 | 1 |
|  | EC VS NEC |  |  | 1 |  |  | 0.65 |  |  | 0.4 |  |  | 0.83 |  |  | 1 |  |  | 1 |  |  | 1 |  |  | 1 |  |  | 1 |
| RD2 | Cases | 38 | 42.2 |  | 8 | 8.9 |  | 22 | 24.4 |  | 7 | 7.8 |  | 3 | 3.3 |  | 3 | 0 |  | 7 | 7.8 |  | 2 | 2.2 |  | 0 | 0 |  |
|  | EC | 42 | 46.7 | 0.55 | 5 | 5.6 | 0.14 | 28 | 31.1 | 0.32 | 6 | 6.7 | 0.77 | 2 | 2.2 | 1 | 2 | 0 | 1 | 5 | 5.6 | 0.77 | 0 | 0 | 0.5 | 0 | 0 | 1 |
|  | NEC | 41 | 45.6 | 0.65 | 3 | 3.3 | 0.21 | 30 | 33.3 | 0.19 | 8 | 8.9 | 0.79 | 1 | 1.1 | 0.62 | 0 | 0 | 0.25 | 5 | 5.6 | 0.55 | 2 | 2.2 | 1 | 0 | 0 | 1 |
|  | EC VS NEC |  |  | 0.72 |  |  | 0.88 |  |  | 0.75 |  |  | 0.58 |  |  | 1 |  |  | 0.5 |  |  | 1 |  |  | 1 |  |  | 1 |
| RD3 | Cases | 71 | 78.9 |  | 0 | 0 |  | 12 | 13.3 |  | 2 | 2.2 |  | 2 | 2.2 |  | 0 | 0 |  | 1 | 1.1 |  | 2 | 2.2 |  | 0 | 0 |  |
|  | EC | 73 | 81.1 | 0.71 | 0 | 0 | 1 | 13 | 14.4 | 0.83 | 2 | 2.2 | 1 | 1 | 1.1 | 1 | 0 | 0 | 1 | 1 | 1.1 | 1 | 0 | 0 | 0.5 | 0 | 0 | 1 |
|  | NEC | 62 | 69.7 | 0.16 | 0 | 0 | 1 | 14 | 15.7 | 0.65 | 3 | 3.4 | 0.68 | 1 | 1.1 | 1 | 1 | 1.1 | 1 | 7 | 7.9 | 0.03* | 1 | 1.1 | 1 | 0 | 0 | 1 |
|  | EC VS NEC |  |  | 1 |  |  | 0.08 |  |  | 0.81 |  |  | 0.68 |  |  | 1 |  |  | 1 |  |  | 0.03* |  |  | 1 |  |  | 1 |
| RD4 | Cases | 35 | 38.9 |  | 0 | 0 |  | 49 | 54.4 |  | 2 | 2.2 |  | 4 | 4.4 |  | 0 | 0 |  | 0 | 0 |  | 0 | 0 |  | 0 | 0 |  |
|  | EC | 41 | 45.6 | 0.37 | 0 | 0 | 1 | 48 | 53.3 | 0.88 | 0 | 0 | 0.5 | 1 | 1.1 | 0.37 | 0 | 0 | 1 | 0 | 0 | 1 | 0 | 0 | 1 | 0 | 0 | 1 |
|  | NEC | 38 | 42.2 | 0.65 | 1 | 1.1 | 1 | 45 | 50 | 0.77 | 1 | 1.1 | 1 | 3 | 3.3 | 1 | 0 | 0 | 1 | 2 | 2.2 | 0.5 | 0 | 0 | 1 | 0 | 0 | 1 |
|  | EC VS NEC |  |  | 1 |  |  | 0.65 |  |  | 0.65 |  |  | 1 |  |  | 0.62 |  |  | 1 |  |  | 0.5 |  |  | 1 |  |  | 1 |
| RD5 | Cases | 77 | 85.6 |  | 0 | 0 |  | 9 | 10 |  | 2 | 2.2 |  | 2 | 2.2 |  | 0 | 0 |  | 0 | 0 |  | 0 | 0 |  | 0 | 0 |  |
|  | EC | 68 | 75.6 | 0.08 | 0 | 0 | 1 | 18 | 20 | 0.06 | 0 | 0 | 0.5 | 2 | 2.2 | 1 | 0 | 0 | 1 | 2 | 2.2 | 0.5 | 0 | 0 | 1 | 0 | 0 | 1 |
|  | NEC | 77 | 86.5 | 0.85 | 0 | 0 | 1 | 9 | 10.1 | 0.98 | 0 | 0 | 0.5 | 2 | 2.2 | 1 | 0 | 0 | 1 | 1 | 1.1 | 0.5 | 0 | 0 | 1 | 0 | 0 | 1 |
|  | EC VS NEC |  |  | 1 |  |  | 0.06 |  |  | 0.06 |  |  | 1 |  |  | 1 |  |  | 0.5 |  |  | 1 |  |  | 1 |  |  | 1 |
| LD1 | Cases | 48 | 53.3 |  | 1 | 1.1 |  | 17 | 18.9 |  | 17 | 18.9 |  | 0 | 0 |  | 0 | 0 |  | 7 | 7.8 |  | 0 | 0 |  | 0 | 0 |  |
|  | EC | 36 | 40 | 0.07 | 0 | 0 | 1 | 32 | 35.6 | 0.01* | 18 | 20 | 0.85 | 0 | 0 | 1 | 0 | 0 | 1 | 4 | 4.4 | 0.54 | 0 | 0 | 1 | 0 | 0 | 1 |
|  | NEC | 42 | 46.7 | 0.37 | 21 | 23.3 | 0.47 | 20 | 22.2 | 0.58 | 21 | 23.3 | 0.47 | 2 | 2.2 | 0.5 | 2 | 2.2 | 1 | 3 | 3.3 | 0.33 | 0 | 0 | 1 | 0 | 0 | 1 |
|  | EC VS NEC |  |  | 0.37 |  |  | 1 |  |  | 0.05 |  |  | 0.59 |  |  | 0.5 |  |  | 1 |  |  | 1 |  |  | 1 |  |  | 1 |
| LD2 | Cases | 35 | 38.9 |  | 12 | 13.3 |  | 20 | 22.2 |  | 7 | 7.8 |  | 7 | 7.8 |  | 3 | 0 |  | 4 | 4.4 |  | 2 | 2.2 |  | 0 | 0 |  |
|  | EC | 31 | 34.8 | 0.57 | 10 | 11.2 | 0.06 | 31 | 34.8 | 0.06 | 5 | 5.6 | 0.77 | 2 | 2.2 | 0.17 | 1 | 0 | 0.62 | 8 | 9 | 0.25 | 1 | 1.1 | 1 | 0 | 0 | 1 |
|  | NEC | 34 | 38.2 | 0.92 | 3 | 3.4 | 0.33 | 36 | 40.4 | 0.01* | 3 | 3.4 | 0.33 | 0 | 0 | 0.01* | 1 | 1.1 | 0.5 | 7 | 7.9 | 0.37 | 1 | 1.1 | 1 | 0 | 0 | 1 |
|  | EC VS NEC |  |  | 0.64 |  |  | 0.44 |  |  | 0.44 |  |  | 0.72 |  |  | 0.5 |  |  | 1 |  |  | 0.79 |  |  | 1 |  |  | 1 |
| LD3 | Cases | 59 | 66.3 |  | 0 | 0 |  | 17 | 19.1 |  | 6 | 6.7 |  | 1 | 1.1 |  | 0 | 0 |  | 4 | 4.5 |  | 2 | 2.2 |  | 0 | 0 |  |
|  | EC | 61 | 67.8 | 0.83 | 2 | 2.2 | 1 | 19 | 21.1 | 0.74 | 1 | 1.1 | 0.06 | 1 | 1.1 | 1 | 0 | 0 | 1 | 5 | 5.6 | 1 | 0 | 0 | 0.25 | 1 | 1.1 | 1 |
|  | NEC | 57 | 63.3 | 0.68 | 0 | 0 | 1 | 24 | 26.7 | 0.23 | 0 | 0 | 0.01* | 1 | 1.1 | 1 | 0 | 0 | 1 | 6 | 6.7 | 0.75 | 1 | 1.1 | 0.62 | 0 | 0 | 1 |
|  | EC VS NEC |  |  | 0.53 |  |  | 1 |  |  | 0.38 |  |  | 1 |  |  | 1 |  |  | 1 |  |  | 1 |  |  | 1 |  |  | 1 |
| LD4 | Cases | 38 | 42.2 |  | 0 | 0 |  | 42 | 46.7 |  | 3 | 3.3 |  | 4 | 4.4 |  | 2 | 0 |  | 0 | 0 |  | 1 | 1.1 |  | 0 | 0 |  |
|  | EC | 36 | 40 | 0.76 | 0 | 0 | 1 | 48 | 53.3 | 0.37 | 0 | 0 | 0.25 | 5 | 5.6 | 1 | 0 | 0 | 0.5 | 1 | 1.1 | 1 | 0 | 0 | 1 | 0 | 0 | 1 |
|  | NEC | 47 | 52.2 | 0.18 | 1 | 1.1 | 0.25 | 33 | 36.7 | 0.17 | 1 | 1.1 | 0.25 | 7 | 7.8 | 0.54 | 0 | 0 | 0.5 | 2 | 2.2 | 0.5 | 0 | 0 | 1 | 0 | 0 | 1 |
|  | EC VS NEC |  |  | 0.1 |  |  | 1 |  |  | 0.02* |  |  | 1 |  |  | 0.77 |  |  | 1 |  |  | 1 |  |  | 1 |  |  | 1 |
| LD5 | Cases | 73 | 81.1 |  | 0 | 0 |  | 12 | 13.3 |  | 2 | 2.2 |  | 3 | 3.3 |  | 0 | 0 |  | 0 | 0 |  | 0 | 0 |  | 0 | 0 |  |
|  | EC | 60 | 66.7 | 0.03* | 1 | 1.1 | 1 | 22 | 24.4 | 0.06 | 4 | 4.4 | 0.68 | 1 | 1.1 | 0.62 | 0 | 0 | 1 | 2 | 2.2 | 0.5 | 0 | 0 | 1 | 0 | 0 | 1 |
|  | NEC | 68 | 76.4 | 0.58 | 1 | 1.1 | 1 | 14 | 15.6 | 0.67 | 1 | 1.1 | 1 | 4 | 4.4 | 1 | 0 | 0 | 1 | 1 | 1.1 | 1 | 0 | 0 | 1 | 0 | 0 | 1 |
|  | EC VS NEC |  |  | 0.1 |  |  | 1 |  |  | 0.14 |  |  | 0.37 |  |  | 0.37 |  |  | 1 |  |  | 1 |  |  | 1 |  |  | 1 |
| *D digit, R* right, *L* left, *EC* endemic control, *NEC* non endemic control, *N* number of values, *** significant values | | | | | | | | | | | | | | | | | | | | | | | | | | | | |
