# Supplementary material for: Qualitative and quantitative dermatoglyphics of chronic kidney disease of unknown origin (CKDu) in Sri Lanka
Source: J Physiol Anthropol. 2020 Jan 17;39:1. doi: 10.1186/s40101-019-0207-0 (PMC6967092; doi:10.1186/s40101-019-0207-0)
Supplement: Supplementary file 3 — Additional file 3: Table S3. Palmar dermatoglyphics (loops) of males. [file 40101_2019_207_MOESM3_ESM.docx]

| **Table S3** Palmar dermatoglyphics (loops) of males | | | | | | | | | | |
| --- | --- | --- | --- | --- | --- | --- | --- | --- | --- | --- |
|  | PL | Cases | | EC | | P1 | NEC | | P2 | P3 |
|  |  | N | % | N | % |  | N | % |  |  |
| Right hand | I | 4 | 4.4 | 1 | 1.1 | 0.37 | 6.7 | 6 | 0.747 | 0.12 |
|  | î | 0 | 0 | 0 | 0 | 1 | 0 | 0 | 1 | 1 |
|  | I^r^ | 7 | 7.8 | 3 | 3.4 | 0.33 | 10 | 9 | 0.6 | 0.13 |
|  | II | 2 | 2.2 | 8 | 9 | 0.06 | 15.6 | 14 | 0 | 0.18 |
|  | îî | 0 | 0 | 0 | 0 | 1 | 0 | 0 | 1 | 1 |
|  | II^T^ | 0 | 0 | 0 | 0 | 1 | 0 | 0 | 1 | 1 |
|  | III | 46 | 51.1 | 47 | 52.8 | 0.82 | 51.1 | 46 | 1 | 0.82 |
|  | îîî | 0 | 0 | 0 | 0 | 1 | 0 | 0 | 1 | 1 |
|  | III^T^ | 13 | 14.4 | 17 | 19.1 | 0.4 | 16.7 | 15 | 0.68 | 0.67 |
|  | IV | 44 | 48.9 | 37 | 41.6 | 0.33 | 46.7 | 42 | 0.77 | 0.49 |
|  | îV | 0 | 0 | 0 | 0 | 1 | 0 | 0 | 1 | 1 |
|  | IV^T^ | 0 | 0 | 0 | 0 | 1 | 0 | 0 | 1 | 1 |
|  | IV^u^ | 0 | 0 | 0 | 0 | 1 | 0 | 0 | 1 | 1 |
|  | H | 12 | 13.3 | 8 | 9 | 0.36 | 14.4 | 13 | 0.83 | 0.26 |
|  | Ĥ | 8 | 8.9 | 7 | 7.9 | 0.8 | 6.7 | 6 | 0.58 | 0.76 |
|  | H^r^ | 0 | 0 | 0 | 0 | 1 | 0 | 0 | 1 | 1 |
|  | T^c^ | 0 | 0 | 0 | 0 | 1 | 0 | 0 | 1 | 1 |
|  | T^r^ | 0 | 0 | 0 | 0 | 1 | 0 | 0 | 1 | 1 |
|  | T^u^ | 0 | 0 | 0 | 0 | 1 | 0 | 0 | 1 | 1 |
| Left hand | I |  | 2.2 | 9 | 10 | 0.06 | 5 | 5.6 | 0.44 | 0.4 |
|  | î | 0 | 0 | 0 | 0 | 1 | 0 | 0 | 1 | 1 |
|  | I^r^ | 6 | 6.7 | 13 | 14.4 | 0.09 | 11 | 12.2 | 0.2 | 0.66 |
|  | II | 0 | 0 | 0 | 0 | 1 | 3 | 3.3 | 0.25 | 0.25 |
|  | îî | 0 | 0 | 0 | 0 | 1 | 0 | 0 | 1 | 1 |
|  | II^T^ | 0 | 0 | 0 | 0 | 1 | 0 | 0 | 1 | 1 |
|  | III | 27 | 30 | 19 | 21.1 | 0.17 | 20 | 22.2 | 0.23 | 0.86 |
|  | îîî | 0 | 0 | 0 | 0 | 1 | 0 | 0 | 1 | 1 |
|  | III^T^ | 19 | 21.1 | 24 | 26.7 | 0.38 | 16 | 17.8 | 0.57 | 0.15 |
|  | IV | 58 | 64.4 | 54 | 60 | 0.54 | 62 | 68.9 | 0.53 | 0.21 |
|  | îV | 0 | 0 | 4 | 4.4 | 0.12 | 1 | 1.1 | 1 | 0.37 |
|  | IV^T^ | 0 | 0 | 0 | 0 | 1 | 1 | 1.1 | 1 | 1 |
|  | IV^u^ | 0 | 0 | 0 | 0 | 1 | 0 | 0 | 1 | 1 |
|  | H | 9 | 10 | 9 | 10 | 1 | 4 | 4.4 | 0.25 | 0.25 |
|  | Ĥ | 8 | 8.9 | 11 | 12.2 | 0.47 | 5 | 5.6 | 0.39 | 0.19 |
|  | H^r^ | 0 | 0 | 0 | 0 | 1 | 0 | 0 | 1 | 1 |
|  | T^c^ | 0 | 0 | 0 | 0 | 1 | 0 | 0 | 1 | 1 |
|  | T^r^ | 0 | 0 | 0 | 0 | 1 | 0 | 0 | 1 | 1 |
|  | T^u^ | 0 | 0 | 0 | 0 | 1 | 0 | 0 | 1 | 1 |
| *PL* Palmar loop, *EC* endemic control, *NEC* non endemic control, *P1* P value of Cases Vs endemic control, *P2* P value of Cases Vs non endemic control, *P3* P value of endemic control Vs non endemic control, *N* number of values, * significant values | | | | | | | | | | |
