# Supplementary material for: Qualitative and quantitative dermatoglyphics of chronic kidney disease of unknown origin (CKDu) in Sri Lanka
Source: J Physiol Anthropol. 2020 Jan 17;39:1. doi: 10.1186/s40101-019-0207-0 (PMC6967092; doi:10.1186/s40101-019-0207-0)
Supplement: Supplementary file 4 — Additional file 4: Table S4. Palmar dermatoglyphics (triradii) of males. [file 40101_2019_207_MOESM4_ESM.docx]

| **Table S4** Palmar dermatoglyphics (triradii) of males | | | | | | | | | | |
| --- | --- | --- | --- | --- | --- | --- | --- | --- | --- | --- |
|  | PT | Cases | | EC | | P1 | NEC | | P2 | P3 |
|  |  | N | % | N | % |  | N | % |  |  |
| Right hand | A | 90 | 100.0 | 89 | 100.0 | 1 | 100 | 90.0 | 1 | 1 |
|  | B | 90 | 100.0 | 89 | 100.0 | 1 | 100 | 90.0 | 1 | 1 |
|  | C | 87 | 96.7 | 87 | 97.8 | 1 | 100 | 90.0 | 0.25 | 0.25 |
|  | D | 90 | 100.0 | 85 | 95.5 | 0.06 | 100 | 90.0 | 1 | 0.06 |
|  | a^1^ | 0 | 0.0 | 5 | 6.0 | 0.03* | 8 | 8.9 | 0.01* | 0.37 |
|  | b^1^ | 2 | 2.2 | 0 | 0.0 | 0.5 | 0 | 0.0 | 0.5 | 1 |
|  | c^1^ | 17 | 18.9 | 12 | 13.5 | 0.33 | 12 | 13.3 | 0.31 | 1 |
|  | d^1^ | 0 | 0.0 | 0 | 0.0 | 1 | 0 | 0.0 | 1 | 0.98 |
|  | t | 77 | 85.6 | 76 | 85.4 | 0.98 | 80 | 72.0 | 0.32 | 0.34 |
|  | t^1^ | 16 | 17.8 | 14 | 15.7 | 0.71 | 19 | 21.1 | 0.57 | 0.35 |
|  | t^11^ | 6 | 6.7 | 6 | 6.7 | 0.98 | 7 | 7.8 | 0.77 | 0.79 |
|  | t^111^ | 2 | 2.2 | 1 | 1.1 | 1 | 0 | 0.0 | 0.5 | 1 |
|  | e | 6 | 6.7 | 2 | 2.2 | 0.28 | 7 | 7.8 | 0.77 | 0.17 |
|  | f | 0 | 0.0 | 2 | 2.2 | 0.25 | 4 | 4.4 | 0.12 | 0.68 |
|  | t^b^ | 10 | 11.1 | 10 | 11.2 | 0.98 | 8 | 8.9 | 0.62 | 0.6 |
|  | t^r^ | 0 | 0.0 | 0 | 0.0 | 1 | 0 | 0 | 1 | 1 |
|  | t^u^ | 0 | 0.0 | 0 | 0.0 | 1 | 2 | 2.2 | 0.5 | 0.5 |
|  | z | 0 | 0.0 | 0 | 0.0 | 1 | 0 | 0.0 | 1 | 1 |
|  | z^1^ | 3 | 3.3 | 2 | 2.2 | 1 | 0 | 0.0 | 1 | 0.25 |
|  | Z^11^ | 3 | 3.3 | 0 | 0.0 | 0.25 | 0 | 0.0 | 1 | 1 |
| Left hand | A | 90 | 100.0 | 90 | 100.0 | 1 | 90 | 100.0 | 1 | 1 |
|  | B | 90 | 100.0 | 90 | 100.0 | 1 | 90 | 100.0 | 1 | 1 |
|  | C | 85 | 94.4 | 84 | 93.3 | 1 | 86 | 95.6 | 1 | 0.75 |
|  | D | 87 | 96.7 | 90 | 100.0 | 0.25 | 88 | 97.8 | 1 | 0.5 |
|  | a^1^ | 0 | 0.0 | 0 | 0.0 | 1 | 1 | 1.1 | 1 | 1 |
|  | b^1^ | 2 | 2.2 | 0 | 0.0 | 0.5 | 0 | 0.0 | 0.5 | 1 |
|  | c^1^ | 22 | 24.4 | 25 | 27.8 | 0.61 | 22 | 24.4 | 1 | 0.61 |
|  | d^1^ | 0 | 0.0 | 0 | 0.0 | 1 | 0 | 0.0 | 1 | 1 |
|  | t | 80 | 88.9 | 78 | 86.7 | 0.65 | 64 | 71.1 | 0.001* | 0.01* |
|  | t^1^ | 10 | 11.1 | 8 | 8.9 | 0.62 | 15 | 16.7 | 0.28 | 0.12 |
|  | t^11^ | 4 | 4.4 | 9 | 10.0 | 0.25 | 6 | 6.7 | 0.75 | 0.42 |
|  | t^111^ | 4 | 4.4 | 1 | 1.1 | 0.37 | 1 | 1.1 | 0.37 | 1 |
|  | e | 6 | 6.7 | 9 | 10.0 | 0.42 | 10 | 11.1 | 0.29 | 0.81 |
|  | f | 2 | 2.2 | 7 | 7.8 | 0.17 | 8 | 8.9 | 0.1 | 0.79 |
|  | t^b^ | 9 | 10.0 | 11 | 12.2 | 0.64 | 6 | 6.7 | 0.42 | 0.2 |
|  | t^r^ | 0 | 0.0 | 0 | 0.0 | 1 | 0 | 0.0 | 1 | 1 |
|  | t^u^ | 0 | 0.0 | 1 | 1.1 | 1 | 0 | 0.0 | 1 | 1 |
|  | z | 0 | 0.0 | 0 | 0.0 | 1 | 0 | 0.0 | 1 | 1 |
|  | z^1^ | 5 | 5.6 | 6 | 6.7 | 1 | 4 | 4.4 | 1 | 0.75 |
|  | Z^11^ | 3 | 3.3 | 0 | 0.0 | 0.25 | 2 | 2.2 | 1 | 0.5 |
| *PT* Palmar triradii, *EC* endemic control, *NEC*  non endemic control, *P1* P value of Cases Vs endemic control, *P2*  P value of Cases Vs non endemic control, *P3* P value of endemic control Vs non endemic control, * significant values | | | | | | | | | | |
