# Supplementary material for: Qualitative and quantitative dermatoglyphics of chronic kidney disease of unknown origin (CKDu) in Sri Lanka
Source: J Physiol Anthropol. 2020 Jan 17;39:1. doi: 10.1186/s40101-019-0207-0 (PMC6967092; doi:10.1186/s40101-019-0207-0)
Supplement: Supplementary file 5 — Additional file 5: Table S5. Palmar dermatoglyphics (loops) of females. [file 40101_2019_207_MOESM5_ESM.docx]

| **Table S5** Palmar dermatoglyphics (loops) of females | | | | | | | | | | |
| --- | --- | --- | --- | --- | --- | --- | --- | --- | --- | --- |
|  | PL | Cases | | EC | | P1 | NEC | | P2 | P3 |
|  |  | N | % | N | % |  | N | % |  |  |
| Right hand | I | 3 | 3.3 | 6 | 6.7 | 0.5 | 8 | 8.9 | 0.21 | 0.58 |
|  | î | 0 | 0.0 | 0 | 0.0 | 1 | 0 | 0.0 | 1 | 1 |
|  | I^r^ | 5 | 5.6 | 8 | 8.9 | 0.57 | 10 | 11.1 | 0.18 | 0.8 |
|  | II | 4 | 4.4 | 8 | 8.9 | 0.37 | 0 | 0.0 | 0.12 | 0.01* |
|  | îî | 0 | 0.0 | 0 | 0.0 | 1 | 3 | 3.3 | 0.25 | 0.25 |
|  | II^T^ | 0 | 0.0 | 0 | 0.0 | 1 | 0 | 0.0 | 1 | 1 |
|  | III | 39 | 43.3 | 46 | 51.1 | 0.3 | 48 | 53.3 | 0.18 | 0.77 |
|  | îîî | 0 | 0.0 | 0 | 0.0 | 1 | 0 | 0.0 | 1 | 1 |
|  | III^T^ | 16 | 17.8 | 10 | 11.1 | 0.2 | 7 | 7.8 | 0.04* | 0.44 |
|  | IV | 53 | 58.9 | 47 | 52.2 | 0.37 | 44 | 48.9 | 0.18 | 0.65 |
|  | îV | 0 | 0.0 | 0 | 0.0 | 1 | 0 | 0.0 | 1 | 1 |
|  | IV^T^ | 0 | 0.0 | 1 | 1.1 | 1 | 0 | 0.0 | 1 | 1 |
|  | IV^u^ | 0 | 0.0 | 1 | 1.1 | 1 | 0 | 0.0 | 1 | 1 |
|  | H | 13 | 14.4 | 11 | 12.2 | 0.66 | 6 | 6.7 | 0.09 | 0.2 |
|  | Ĥ | 13 | 14.4 | 16 | 17.8 | 0.54 | 6 | 6.7 | 0.09 | 0.02* |
|  | H^r^ | 1 | 1.1 | 1 | 1.1 | 1 | 1 | 1.1 | 1 | 1 |
|  | T^c^ | 0 | 0.0 | 0 | 0.0 | 1 | 0 | 0.0 | 1 | 1 |
|  | T^r^ | 0 | 0.0 | 0 | 0.0 | 1 | 0 | 0.0 | 1 | 1 |
|  | T^u^ | 0 | 0.0 | 0 | 0.0 | 1 | 0 | 0.0 | 1 | 1 |
| Left hand | I | 5 | 5.6 | 8 | 8.9 | 0.57 | 10 | 11.1 | 0.18 | 0.62 |
|  | î | 0 | 0.0 | 1 | 1.1 | 1 | 0 | 0.0 | 1 | 1 |
|  | I^r^ | 7 | 7.8 | 11 | 12.2 | 0.26 | 10 | 11.1 | 0.61 | 0.82 |
|  | II | 1 | 1.1 | 3 | 3.3 | 0.62 | 5 | 5.6 | 0.21 | 0.72 |
|  | îî | 0 | 0.0 | 0 | 0.0 | 1 | 0 | 0.0 | 1 | 1 |
|  | II^T^ | 0 | 0.0 | 0 | 0.0 | 1 | 0 | 0.0 | 1 | 1 |
|  | III | 27 | 30.0 | 30 | 33.3 | 0.63 | 28 | 31.1 | 0.87 | 0.75 |
|  | îîî | 0 | 0.0 | 0 | 0.0 | 1 | 0 | 0.0 | 1 | 1 |
|  | III^T^ | 15 | 16.7 | 11 | 12.2 | 0.4 | 9 | 10.0 | 0.19 | 0.64 |
|  | IV | 59 | 65.6 | 68 | 75.6 | 0.14 | 56 | 62.2 | 0.64 | 0.045* |
|  | îV | 0 | 0.0 | 0 | 0.0 | 1 | 2 | 2.2 | 0.5 | 0.5 |
|  | IV^T^ | 1 | 1.1 | 0 | 0.0 | 1 | 0 | 0.0 | 1 | 1 |
|  | IV^u^ | 0 | 0.0 | 0 | 0.0 | 1 | 0 | 0.0 | 1 | 1 |
|  | H | 11 | 12.2 | 8 | 8.9 | 0.47 | 8 | 8.9 | 0.47 | 1 |
|  | Ĥ | 8 | 8.9 | 12 | 13.3 | 0.34 | 6 | 6.7 | 0.58 | 0.14 |
|  | H^r^ | 0 | 0.0 | 0 | 0.0 | 1 | 0 | 0.0 | 1 | 1 |
|  | T^c^ | 0 | 0.0 | 0 | 0.0 | 1 | 0 | 0.0 | 1 | 1 |
|  | T^r^ | 0 | 0.0 | 0 | 0.0 | 1 | 0 | 0.0 | 1 | 1 |
|  | T^u^ | 0 | 0.0 | 0 | 0.0 | 1 | 0 | 0.0 | 1 | 1 |
| PL Palmar loop, *EC* endemic control, *NEC* non endemic control, *P1* P value of Cases Vs endemic control, *P2* P value of Cases Vs non endemic control, *P3* P value of endemic control Vs non endemic control, *N* number of values, *** significant values | | | | | | | | | | |
