# Supplementary material for: Qualitative and quantitative dermatoglyphics of chronic kidney disease of unknown origin (CKDu) in Sri Lanka
Source: J Physiol Anthropol. 2020 Jan 17;39:1. doi: 10.1186/s40101-019-0207-0 (PMC6967092; doi:10.1186/s40101-019-0207-0)
Supplement: Supplementary file 6 — Additional file 6: Table S6. Palmar dermatoglyphics (triradii) of females. [file 40101_2019_207_MOESM6_ESM.docx]

| **Table S6** Palmar dermatoglyphics (triradii) of females | | | | | | | | | | |  |
| --- | --- | --- | --- | --- | --- | --- | --- | --- | --- | --- | --- |
|  | PT | Cases | | EC | | P1 | NEC | | P2 | P3 | |
|  |  | N | % | N | % |  | N | % |  |  |  |
| Right hand | A | 90 | 100 | 1 | 1.1 | 1 | 90 | 100 | 1 | 1 | |
|  | B | 90 | 100 | 89 | 100 | 1 | 90 | 100 | 1 | 1 | |
|  | C | 89 | 98.9 | 86 | 95.6 | 0.37 | 85 | 94.4 | 0.21 | 1 | |
|  | D | 86 | 95.6 | 90 | 100 | 0.12 | 89 | 98.9 | 0.37 | 1 | |
|  | a^1^ | 0 | 0 | 6 | 6.7 | 0.03* | 6 | 6.7 | 0.03* | 1 | |
|  | b^1^ | 2 | 2.2 | 0 | 0 | 0.5 | 0 | 0 | 0.5 | 1 | |
|  | c^1^ | 11 | 12.2 | 23 | 25.6 | 0.04* | 20 | 22.2 | 0.08 | 0.6 | |
|  | d^1^ | 0 | 0 | 0 | 0 | 1 | 0 | 0 | 1 | 1 | |
|  | t | 74 | 82.2 | 71 | 78.9 | 0.57 | 70 | 77.8 | 0.46 | 0.86 | |
|  | t^1^ | 16 | 17.8 | 18 | 20 | 0.7 | 19 | 21.1 | 0.57 | 0.85 | |
|  | t^11^ | 12 | 13.3 | 5 | 5.6 | 0.12 | 3 | 3.3 | 0.03* | 0.72 | |
|  | t^111^ | 1 | 1.1 | 2 | 2.2 | 1 | 1 | 1.1 | 1 | 1 | |
|  | e | 5 | 5.6 | 7 | 7.8 | 0.77 | 9 | 10 | 0.4 | 0.6 | |
|  | f | 1 | 1.1 | 3 | 3.3 | 0.62 | 0 | 0 | 1 | 0.25 | |
|  | t^b^ | 11 | 12.2 | 15 | 16.7 | 0.4 | 7 | 7.8 | 0.32 | 0.07 | |
|  | t^r^ | 0 | 0 | 0 | 0 | 1 | 0 | 0 | 1 | 1 | |
|  | t^u^ | 0 | 0 | 1 | 1.1 | 1 | 0 | 0 | 1 | 1 | |
|  | Z | 0 | 0 | 1 | 1.1 | 1 | 0 | 0 | 1 | 1 | |
|  | Z^1^ | 1 | 1.1 | 4 | 4.4 | 0.37 | 5 | 5.6 | 0.21 | 1 | |
|  | Z^11^ | 4 | 4.4 | 0 | 0 | 0.12 | 1 | 1.1 | 0.37 | 1 | |
| Left hand | A | 90 | 100 | 90 | 100 | 1 | 90 | 100 | 1 | 1 | |
|  | B | 90 | 100 | 90 | 100 | 1 | 90 | 100 | 1 | 1 | |
|  | C | 82 | 91.1 | 86 | 95.6 | 0.37 | 82 | 91.1 | 1 | 0.37 | |
|  | D | 89 | 98.9 | 90 | 100 | 1 | 90 | 100 | 1 | 1 | |
|  | a^1^ | 0 | 0 | 2 | 2.2 | 0.5 | 4 | 4.4 | 0.12 | 0.68 | |
|  | b^1^ | 1 | 1.1 | 0 | 0 | 1 | 0 | 0 | 1 | 1 | |
|  | c^1^ | 18 | 20 | 30 | 33.3 | 0.04* | 18 | 20 | 1 | 0.04* | |
|  | d^1^ | 1 | 1.1 | 0 | 0 | 1 | 0 | 0 | 1 | 1 | |
|  | t | 64 | 71.1 | 68 | 75.6 | 0.5 | 65 | 72.2 | 0.87 | 0.61 | |
|  | t^1^ | 25 | 27.8 | 15 | 16.7 | 0.07 | 24 | 26.7 | 0.87 | 0.1 | |
|  | t^11^ | 15 | 16.7 | 9 | 10 | 0.19 | 6 | 6.7 | 0.04* | 0.42 | |
|  | t^111^ | 0 | 0 | 3 | 3.3 | 0.25 | 1 | 1.1 | 1 | 0.62 | |
|  | e | 8 | 8.9 | 11 | 12.2 | 0.47 | 9 | 10 | 0.8 | 0.64 | |
|  | f | 3 | 3.3 | 3 | 3.3 | 1 | 4 | 4.4 | 1 | 1 | |
|  | t^b^ | 9 | 10 | 14 | 15.6 | 0.26 | 6 | 6.7 | 0.15 | 0.06 | |
|  | t^r^ | 0 | 0 | 0 | 0 | 1 | 0 | 0 | 1 | 1 | |
|  | t^u^ | 1 | 1.1 | 0 | 0 | 1 | 0 | 0 | 1 | 1 | |
|  | Z | 0 | 0 | 0 | 0 | 1 | 0 | 0 | 1 | 1 | |
|  | Z^1^ | 8 | 8.9 | 4 | 4.4 | 0.37 | 8 | 8.9 | 1 | 0.37 | |
|  | Z^11^ | 1 | 1.1 | 0 | 0 | 1 | 0 | 0 | 1 | 1 | |
| *PT* Palmar triradii, *EC* endemic control, *NEC* non endemic control, *P1* P value of Cases Vs endemic control, *P2* P value of Cases Vs non endemic control, *P3* P value of endemic control Vs non endemic control, *N* number of values, *** significant values | | | | | | | | | | |  |
