# Supplementary material for: Qualitative and quantitative dermatoglyphics of chronic kidney disease of unknown origin (CKDu) in Sri Lanka
Source: J Physiol Anthropol. 2020 Jan 17;39:1. doi: 10.1186/s40101-019-0207-0 (PMC6967092; doi:10.1186/s40101-019-0207-0)
Supplement: Supplementary file 7 — Additional file 7: Table S7. Directional asymmetry. [file 40101_2019_207_MOESM7_ESM.docx]

**Table S7** Directional asymmetry

|  |  | Cases | | Endemic control | | Non endemic control | |
| --- | --- | --- | --- | --- | --- | --- | --- |
|  | Variable | F | P | F | P | F | P |
|  | D1 RC | 2.41 | 0.12 | 7.75 | 0.01 | 6.83 | 0.01 |
|  | D2 RC | 0.03 | 0.87 | 0.11 | 0.74 | 0.56 | 0.46 |
|  | D3 RC | 1.31 | 0.26 | 0.08 | 0.78 | 0.33 | 0.57 |
|  | D4 RC | 1.21 | 0.27 | 0.50 | 0.48 | 2.07 | 0.15 |
|  | D5 RC | 1.30 | 0.26 | 0.38 | 0.54 | 1.15 | 0.28 |
|  | TRC | 1.52 | 0.22 | 0.88 | 0.35 | 1.49 | 0.22 |
|  | A-B RC | 1.61 | 0.21 | 2.11 | 0.15 | 2.07 | 0.15 |
| Male | D1 RC | 5.18 | 0.02 | 3.22 | 0.07 | 3.08 | 0.08 |
|  | D2 RC | 0.19 | 0.67 | 0.00 | 0.96 | 0.55 | 0.46 |
|  | D3 RC | 2.15 | 0.15 | 0.13 | 0.72 | 0.79 | 0.37 |
|  | D4 RC | 0.39 | 0.54 | 0.06 | 0.82 | 0.06 | 0.82 |
|  | D5 RC | 0.01 | 0.92 | 0.01 | 0.95 | 1.73 | 0.19 |
|  | TRC | 1.77 | 0.19 | 0.14 | 0.71 | 1.45 | 0.23 |
|  | A-B RC | 0.66 | 0.42 | 0.00 | 0.98 | 1.28 | 0.26 |
| *A-B RC* a-b Ridge count, *TRC* total ridge count, *D* digit , *RC* ridge count, *F* and *P* values for the random effect ‘individual’, and fixed effect side (e.g. right or left) (factorial ANOVA), * significant values | | | | | | | |
